# ETS-4 Is a Transcriptional Regulator of Life Span in *Caenorhabditis elegans*

**DOI:** 10.1371/journal.pgen.1001125

**Published:** 2010-09-16

**Authors:** Bargavi Thyagarajan, Adam G. Blaszczak, Katherine J. Chandler, Jennifer L. Watts, W. Evan Johnson, Barbara J. Graves

**Affiliations:** 1 Huntsman Cancer Institute, Department of Oncological Sciences, University of Utah, Salt Lake City, Utah, United States of America; 2 School of Molecular Biosciences, Washington State University, Pullman, Washington, United States of America; 3 Department of Statistics, Brigham Young University, Provo, Utah, United States of America; University of California San Francisco, United States of America

## Abstract

Aging is a complex phenotype responsive to a plethora of environmental inputs; yet only a limited number of transcriptional regulators are known to influence life span. How the downstream expression programs mediated by these factors (or others) are coordinated into common or distinct set of aging effectors is an addressable question in model organisms, such as *C. elegans*. Here, we establish the transcription factor ETS-4, an ortholog of vertebrate *SPDEF*, as a longevity determinant. Adult worms with *ets-4* mutations had a significant extension of mean life span. Restoring ETS-4 activity in the intestine, but not neurons, of *ets-4* mutant worms rescued life span to wild-type levels. Using RNAi, we demonstrated that *ets-4* is required post-developmentally to regulate adult life span; thus uncoupling the role of ETS-4 in aging from potential functions in worm intestinal development. Seventy ETS-4-regulated genes, identified by gene expression profiling of two distinct *ets-4* alleles and analyzed by bioinformatics, were enriched for known longevity effectors that function in lipid transport, lipid metabolism, and innate immunity. Putative target genes were enriched for ones that change expression during normal aging, the majority of which are controlled by the GATA factors. Also, some ETS-4-regulated genes function downstream of the FOXO factor, DAF-16 and the insulin/IGF-1 signaling pathway. However, epistasis and phenotypic analyses indicate that *ets-4* functioned in parallel to the insulin/IGF-1 receptor, *daf-2* and *akt-1/2* kinases. Furthermore, *ets-4* required *daf-16* to modulate aging, suggesting overlap in function at the level of common targets that affect life span. In conclusion, ETS-4 is a new transcriptional regulator of aging, which shares transcriptional targets with GATA and FOXO factors, suggesting that overlapping pathways direct common sets of lifespan-related genes.

## Author Summary

Animal life span is regulated in response to developmental and environmental inputs through coordinate changes in gene expression. Thus, longevity determinants include DNA-binding proteins that regulate gene expression by controlling transcription. Here, we explored the physiological role of the transcriptional regulator, ETS-4, in the roundworm *Caenorhabditis elegans*. Our data showed that worms that lack ETS-4 lived significantly longer, revealing ETS-4′s role in the transcription network that regulates life span. We identified 70 genes whose expression was modulated by ETS-4 that function in lipid transport, lipid metabolism and innate immunity. Some of the ETS-4-regulated genes were also controlled by two other regulators of aging, the FOXO and GATA factors. We concluded that a common set of transcriptional targets orchestrate the network of physiological factors that affect aging. ETS-4 is closely related to the human ETS protein SPDEF that exhibits aberrant expression in breast and prostate tumors. Because the genetic pathways that regulate aging are well conserved in other organisms, including humans, our findings could lead to a better understanding of SPDEF function and longevity regulation in mammals.

## Introduction

The emerging picture from studies with model organisms is that animal life span is regulated by coordination of gene regulatory networks in response to environmental inputs. In *C. elegans* a number of transcription factors function as genetic modifiers of aging, including Forkhead (DAF-16 and PHA-4) and GATA (ELT-3, ELT-5, and ELT-6) factors [1]–[3]. The transcription factors that function in life span determination often respond to evolutionarily conserved pathways and cellular processes, including insulin/IGF-1 signaling, c-Jun N-terminal kinase signaling (JNK), the Target of rapamycin pathway (TOR), caloric intake, mitochondrial respiration and signaling from the germ line [1], [2], [4]–[8]. Gene expression profiling has identified overlapping downstream targets for these transcription factors that regulate development, metabolism, reproduction, stress response and innate immunity [3], [9]–[11]. Consistent with this complexity, genetic tests show that disruption of individual downstream target genes has a modest impact on longevity. Therefore, the downstream effectors of each transcription factor are proposed to act collectively to mediate the significant impact of signaling pathways on life span [9].

In this study, we identify the ETS transcription factor, ETS-4, as a longevity determinant in *C. elegans*. Gene knock-out studies performed for 20 of the 26 *ETS* genes in mice have implicated ETS factors in diverse cellular processes such as proliferation, differentiation, migration, apoptosis, and cell-cell interactions [12]–[15]. However, discerning molecular mechanisms of ETS protein function in mice has been complicated by the large number of *ETS* paralogs expressed in any particular cell type [15], [16]. *C. elegans*, with only ten *ets* genes, provides a simpler and more genetically tractable model to investigate ETS factor function *in vivo*.

*C. elegans ets-4* is the apparent ortholog of vertebrate *SPDEF*/SAM pointed domain containing ETS transcription factor (also known as *PDEF*/Prostate derived ETS factor) (Figure S1). The mRNA levels of *SPDEF* are altered in breast and prostate tumors [17], [18]. Studies using tumor cell lines show that SPDEF affects cell migration and invasion pathways [18]–[23]. A deletion allele in mice suggests a role in specialized intestinal epithelial cell differentiation [24]. Thus, SPDEF may have significant disease relevance, yet its physiological role in normal animal development and homeostasis is not completely understood.

To decipher *ets-4* function, we undertook a reverse genetics approach in *C. elegans* and established *ets-4* as a transcriptional regulator of longevity. Expression of *ets-4* was observed in a number of tissues, however, transgenic rescue experiments implicated the intestine in the aging phenotype. Gene expression profiling identified ETS-4-regulated genes that function in life span determination. Strikingly, a significant number of these life span effectors have been previously shown to function downstream of the insulin/IGF-1 signaling pathway as well as the GATA factor, ELT-3. Genetic tests reveal that *ets-4* functions in parallel to the insulin/IGF-1 signaling pathway, yet requires the FOXO transcription factor, *daf-16*, to modulate life span. Taken together, our findings identify the physiological role of ETS-4 in *C. elegans* and indicate transcriptional control of aging effectors by ETS-4.

## Results

### ETS-4 Is a Novel Longevity Determinant

**Figure 1:**
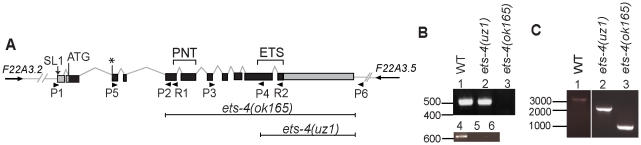
Schematic of *ets-4* gene structure and gene deletion analysis. (A) Gene structure of *ets-4*. Black boxes represent exons linked by lines that indicate introns. The 3′UTR and 5′UTR are shaded gray. The proposed translation start site ATG, confirmed site of addition of the SL1 sequence and exons encoding the ETS and PNT domains are indicated. * denotes the start of an alternative isoform (WormBase web site, http://www.wormbase.org, release WS214). The orientations of neighboring genes are represented by arrows. Sequences deleted in the *ok165* and *uz1* alleles are marked. The position of primers used for reverse transcription (R1, R2) and PCR (P1, P2, P3, P4, P5, P6) analyses are indicated. (B) Agarose gel showing RT-PCR analysis of wild-type (WT), *ets-4(uz1)* and *ets-4(ok165)* worms with primers R1, P1, P2 (top panel) and R2, P3, P4 (bottom panel). DNA size indicated in bp. (C) Agarose gel showing PCR analysis of wild-type (WT), *ets-4(uz1)* and *ets-4(ok165)* worms with primers P5 and P6. DNA size indicated in bp.

To determine the function of ETS-4 *in vivo*, *ets-4(ok165)* worms carrying a deletion at the *ets-4* locus were analyzed. Coding sequences for both the ETS DNA binding domain, and the PNT domain, which is a protein-protein interaction domain conserved in a subset of ETS proteins, were lacking in the *ets-4(ok165)* worms (Figure 1A and 1C). Animals carrying the *ok165* allele lacked full-length *ets-4* mRNA as confirmed by RT-PCR analysis (Figure 1B). Therefore, we propose that *ets-4(ok165)* is a null allele for ETS-4 function.

**Figure 2:**
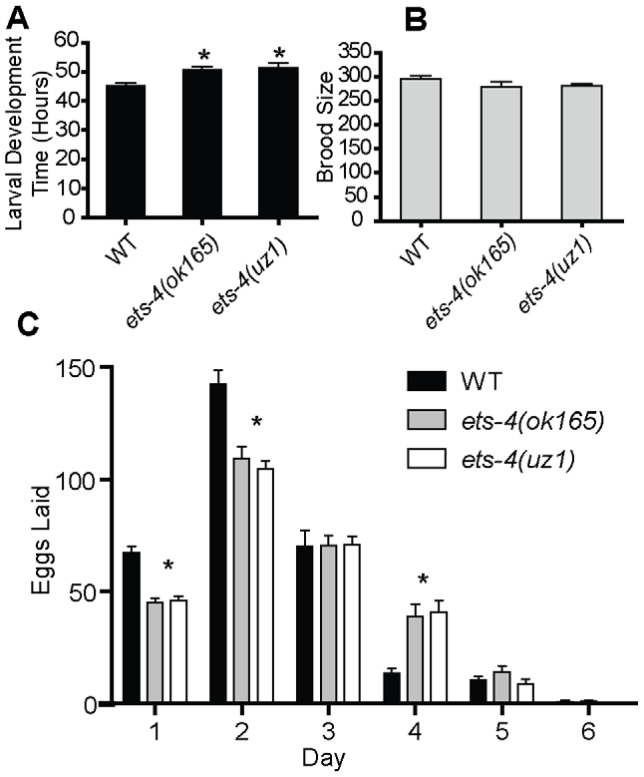
ETS-4 affects larval developmental rate and egg-laying rate. (A) The larval development time (hr) defined as the time from L1 to pre-fertile adult for wild-type (WT), *ets-4(ok165)* and *ets-4(uz1)* worms at 20°C. Values are mean ± SEM and * indicates p<0.05. (B) Total number of progeny (brood size, mean ± SEM) at 20°C was counted for individual WT (n = 11), *ets-4(ok165)* (n = 16) and *ets-4(uz1)* (n = 18) worms. (C) The number of eggs laid (mean ± SEM) during each day of the egg-laying period for WT, *ets-4(ok165)*, and *ets-4(uz1)* worms at 20°C (* indicates p<0.001).

After outcrossing six times to the wild-type (N2) strain, the *ets-4(ok165)* worms were examined for phenotypes. The larval developmental time, measured as the time taken for L1 larvae to reach the young adult stage at 20°C, was 6–8 hr longer in *ets-4(ok165)* than wild-type worms (Figure 2A). Despite this 10–13% delay, *ets-4(ok165)* larvae exhibited apparently wild-type development and morphology. Neither arrests at particular larval stages nor molting defects were observed. The self brood size of *ets-4(ok165)* worms was similar to that of wild-type worms, suggesting normal fecundity (Figure 2B). However, a difference in the rate of egg-laying was observed (Figure 2C). During the peak egg-laying period (day 2 of egg-laying) the *ets-4(ok165)* worms laid significantly fewer eggs (109±5) than wild-type worms (142±6) (Figure 2C). In addition, *ets-4(ok165)* hermaphrodites produced significantly more progeny (39±6) later in life (day 4 of egg-laying) than wild-type worms (14±2) (Figure 2C). The *ets-4(ok165)* males exhibited mating efficiency comparable to wild-type males in crosses with temperature-sensitive *fem-3(e2006)* hermaphrodites [25] that are incapable of producing self-progeny at the restrictive temperature (data not shown). In summary, *ets-4(ok165)* worms exhibited a 10–13% delay in larval development and an altered egg-laying rate, but were wild-type in morphology, development and fecundity suggesting normal fitness.

**Figure 3:**
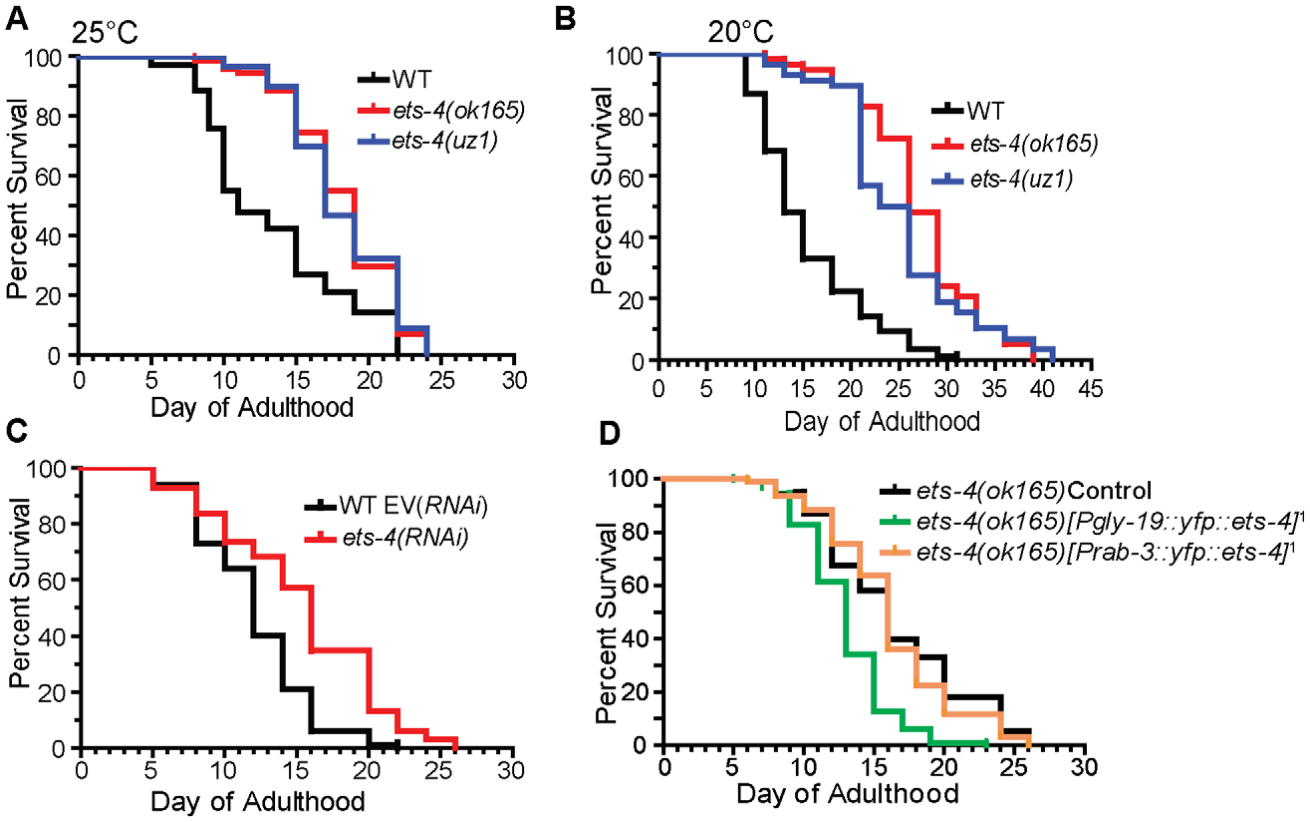
ETS-4 regulates *C. elegans* life span. Survival curves from life span experiments. See Table 1 and Table S1 for data from additional trials, mean life span and statistical analyses. (A) Survival curves of wild-type (WT), *ets-4(ok165)* and *ets-4(uz1)* worms at 25°C. (B) Survival curves of wild-type (WT), *ets-4(ok165)* and *ets-4(uz1)* worms at 20°C. (C) Survival curves for wild-type (WT) worms grown at 25°C and subjected to *ets-4(RNAi)* or an empty vector (EV) control RNAi starting at the L4 stage. (D) Survival curves of *ets-4(ok165)* transgenic animals with tissue-specific expression of *ets-4* in intestinal cells, *ets-4(ok165);[Pgly-19::yfp::ets-4]* ^1^, or in neurons, *ets-4(ok165);[Prab-3::yfp::ets-4]* ^1^. ^1^refers to one of the two independent transgenic lines generated. Injection control strain indicated as *ets-4(ok165)* Control.

Considering a post-developmental role for *ets-4*, we monitored the adult life span of *ets-4(ok165)* worms. Because different laboratory strains of *C. elegans* can have significantly different life spans [26], we compared the life spans of *ets-4(ok165)* animals with those of isogenic *ets-4(+)* controls obtained by outcrossing *ets-4(ok165)* to the wild-type (N2) strain. Age-matched wild-type or *ets-4(ok165)* L4 stage larvae were picked and the first day of adulthood counted as day one. At 25°C, the mean adult life span of *ets-4(ok165)* worms (18.0±0.4 days) was significantly longer than that of isogenic *ets-4(+)* wild-type worms (13.3±0.6 days) (Figure 3A and Table 1). Thus, loss of *ets-4* led to a significantly longer mean adult life span compared to wild-type worms at 25°C (Figure 3A, Table 1 and Table S1). Since growth temperature strongly influences *C. elegans* longevity [27], the life span of *ets-4(ok165)* animals was also monitored at 20°C. The mean adult life span of *ets-4(ok165)* animals at 20°C (27.1±0.8 days) was significantly longer than that of isogenic *ets-4(+)* wild-type worms (15.4±0.6 days), confirming the extended life span phenotype of *ets-4* null mutant animals (Figure 3B and Table 1). Because feeding defective (*eat*) mutant animals are also slow-growing and long-lived [6], [28], the feeding behavior of *ets-4(ok165)* was examined by recording the pharyngeal pumping and defecation rates. Under well-fed conditions, the feeding behavior of *ets-4(ok165)* worms was indistinguishable from that of wild-type animals (Figure S2). Thus, ETS-4 regulates life span without modifying feeding behavior.

**Table 1:** Summary of Life Span Analysis for *ets-4(ok165)* and *ets-4(uz1)* Worms^a^.

Genotype^b^	Mean Life Span±SEM^b^ (Days)	N^b^,^c^	p-value^b^ Vs WT controls	Vs Strain^b^	p-value^b^
WT d	13.3±0.6	71			
*ets-4(ok165)*	18.0±0.4	71	<0.0001		
*ets-4(uz1)*	18.2±0.4	90	<0.0001		
WT 20°C	15.4±0.6	85			
*ets-4(ok165)* 20°C	27.1±0.8	78	<0.0001		
*ets-4(uz1)* 20°C	25.0±0.9	68	<0.0001		
WT EV(*RNAi*)^e^	12.0±0.4	100			
*ets-4(RNAi)*	15.3±0.6	98	<0.0001		
WT EV(*RNAi*)	10.0±0.2	39			
*ets-4(ok165)* EV(*RNAi*)	11.6±0.2	64	<0.0001		
*daf-16(RNAi)*	8.4±0.1	62	<0.0001		
*daf-16(RNAi); ets-4(ok165)*	8.4±0.1	63	<0.0001	*ets-4(ok165)*EV(*RNAi*)	<0.0001
				*daf-16(RNAi)*	0.85(ns)^f^
WT EV(*RNAi*)	13.3±0.5	96			
*ets-4(ok165)* EV(*RNAi*)	16.7±0.6	82	<0.0001		
*daf-2(RNAi)*	22.4±1.4	90	<0.0001		
*daf-2(RNAi); ets-4(ok165)*	29.0±1.6	51	<0.0001	*ets-4(ok165)* EV(*RNAi*)	<0.0001
				*daf-2(RNAi)*	<0.0001
*akt-1/2(RNAi)*	20.4±1.0	65	<0.0001		
*akt-1/2(RNAi); ets-4(ok165)*	24.6±1.0	79	<0.0001	*ets-4(ok165)* EV(*RNAi*)	<0.0001
				*akt-1/2(RNAi)*	<0.0001
WT	11.4±0.3	102			
*ets-4(ok165)*	18.6±0.5	90	<0.0001		
*ets-4(ok165)* Control^g^	16.5±0.5	93			
*ets-4(ok165)[Pgly-19::yfp::ets-4]* 1	12.9±0.3	111		*ets-4(ok165)* Control	<0.0001
*ets-4(ok165)[Pgly-19::yfp::ets-4]* 2	14.4±0.4	90		*ets-4(ok165)* Control	<0.0001
*ets-4(ok165)[Prab-3::yfp::ets-4]* 1	16.1±0.5	94		*ets-4(ok165)* Control	0.42(ns)
*ets-4(ok165)[Prab-3::yfp::ets-4]* 2	17.3±0.6	65		*ets-4(ok165)* Control	0.35(ns)

a Independent repeats of each life span experiment were performed. Data from representative experiments are shown. Data from repeats are shown in Table S1.

b Life span data sets within each panel of this table were done in parallel and statistical analyses was done within the data set.

c Number of worms scored.

d WT refers to wild-type.

e EV refers to empty vector.

f ns indicates p-values>0.05 that were not considered significant.

g *ets-4(ok165)* Control refers to injection control strain. Strain details in Text S1.

1,2 Refer to different independent lines.

To further characterize the role of *ets-4* in life span regulation, we initiated *ets-4* inactivation by RNAi at the L4 larval stage to bypass potential earlier developmental roles [29]. Similar to the long-lived phenotype of *ets-4(ok165)* mutant animals, *ets-4(RNAi)* on wild-type worms resulted in significant extension of mean adult life span (Figure 3C and Table 1). These data suggest that *ets-4* functions post-developmentally in regulation of adult life span.

We generated a second deletion allele of *ets-4*, *uz1*, which removes sequences coding for most of the ETS domain and the 3′UTR, but retains the PNT domain coding sequence (Figure 1A and 1C). An abridged *ets-4* mRNA that could encode a truncated protein was detected in *ets-4(uz1)* worms (Figure 1B). Similar to the *ets-4(ok165)* worms, we observed slow larval growth, altered progeny production and extended life span phenotypes in *ets-4(uz1)* mutant animals (Figure 2, Figure 3A and 3B). Thus, examination of this second *ets-4* deletion allele corroborated the loss-of-function phenotypes observed in *ets-4(ok165)* mutant animals and enabled further use of this allele. However, *ets-4(uz1)* worms exhibited several unique phenotypes not observed in *ets-4(ok165)* worms including ruptured vulva, distorted seam cell syncytia and broken alae (data not shown). We speculate that these added effects are likely due to the interfering activity of the truncated ETS-4 encoded by the *uz1* allele that would lack the ability to bind to ETS-4 target genes and yet, may interact with various protein partners through the PNT domain. Therefore, the additional phenotypes associated with the *ets-4(uz1)* allele may be involved with, but not necessarily restricted to, normal ETS-4 function and were not studied further. Nevertheless, analyses of two distinct deletion alleles of *ets-4* enabled the identification of *ets-4* loss-of-function phenotypes.

### Tissues That Control Life Span Express *ets-4*

**Figure 4:**
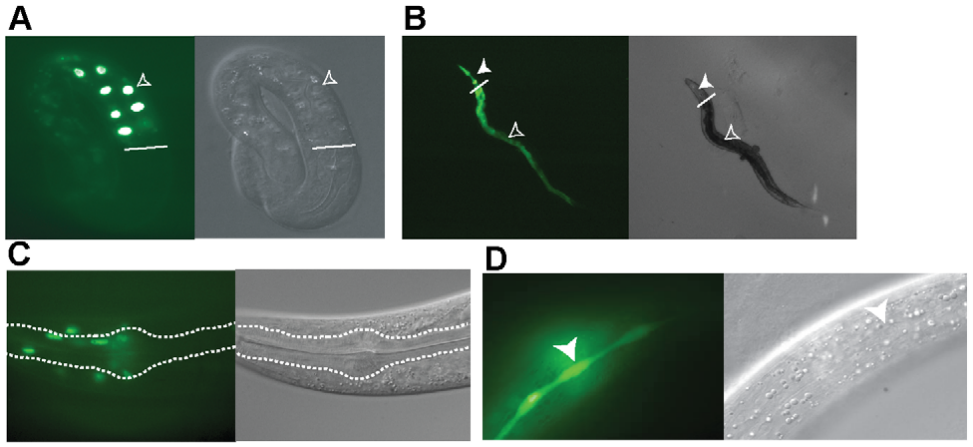
Expression pattern of *ets-4*. Transgenic worms expressing the *Pets-4::gfp* promoter fusion (B and D) or the *ets-4::gfp* promoter and open reading frame construct (A and C) are shown. Differential interference contrast (DIC) and fluorescence images were visualized using a Zeiss Axioskop 2 microscope. (A) GFP expression in the intestinal cells of a 3-fold stage embryo (open arrowhead). White line indicates start of the intestine. (B) GFP expression in the intestinal (open arrowhead) and pharyngeal (filled arrowhead) cells. White line indicates the demarcation between the pharynx and intestine. (C) GFP expression in the cells in and around the pharynx of an adult worm. The procorpus, metacarpus and part of the isthmus are outlined. (D) GFP expression in the seam cell syncytium (filled arrowhead) of an adult worm.

To better understand how ETS-4 regulates life span, we sought to identify the tissues in which ETS-4 functions. GFP reporter constructs containing 5 kb of the *ets-4* promoter alone (*Pets-4::gfp*) or including the genomic DNA coding for ETS-4 (*ets-4::gfp*) were used to generate several independent transgenic lines. Robust GFP expression for both constructs was observed in the intestinal cells of transgenic worms starting at the 3-fold embryonic stage and was maintained through larval development and in the adult (Figure 4A and 4B). GFP expression was also seen in several cells of the anterior and posterior bulbs of the pharynx and in seam cells (Figure 4B, 4C and 4D). Lastly, *ets-4* expression was observed in a few unidentified cells of the vulva, hypodermal nuclei, several unidentified neurons, labial socket cells of the head, and a few cells of the rectum (data not shown). Thus, these results expanded the observations made in previous studies using shorter regions of the *ets-4* promoter in GFP constructs [30]–[32] and demonstrated *ets-4* expression in the intestine and neurons, key tissues known to regulate longevity in worms [33]–[35].

### ETS-4 Functions in the Intestine to Regulate Life Span

Studies in mammals, flies and worms have identified subsets of cells, including adipose, intestinal and neuronal tissues, that affect the rate of aging of the entire organism [33]–[40]. To identify cell types in which ETS-4 functions to modulate life span, a transgene encoding a YFP::ETS-4 fusion was expressed in intestinal cells or neurons of *ets-4(ok165)* worms by use of the *gly-19* or *rab-3* promoters, respectively [35], [41], [42]. Analysis of at least two independent transgenic lines showed that YFP::ETS-4 expressed in intestinal cells restored the life span of *ets-4(ok165)* worms to wild-type (Figure 3D, Table 1 and Table S1). In contrast, the extended life span phenotype of *ets-4(ok165)* worms was not affected by YFP::ETS-4 expression in neurons (Figure 3D, Table 1 and Table S1). Additionally, YFP::ETS-4 expression in either cell type did not rescue the altered egg-laying rate phenotype of *ets-4(ok165)* worms, indicating that the longevity and egg-laying phenotypes were separable (Figure S3). Animals expressing YFP::ETS-4 had wild-type brood sizes, suggesting normal fecundity and fitness (Figure S3). Moreover, because the life span of rescued lines was not shortened beyond wild-type controls, we concluded that YFP::ETS-4 fusion was not toxic in these tissues (Table 1). In summary, ETS-4 functions in the intestine to modulate life span.

### ETS-4 Regulated Genes

Because ETS-4 is implicated to be a transcription factor, a role in life span determination predicts a set of ETS-4 target genes that function in aging. We took advantage of the two distinct strains with a disrupted *ets-4* locus to investigate effects on gene expression. We chose late L4 stage larvae because of ease in staging and relevance to adult intestine function. Microarray-based expression profiling of wild-type and *ets-4(ok165)* larvae identified 145 genes whose expression was altered with 88 genes down-regulated 2.2 fold or more (Table S2). qRT-PCR analyses in age-matched, one-day old adults confirmed the differential expression observed for nine out of nine genes selected randomly from the top-thirty changed genes (Figure S4). As predicted by the broader phenotypic consequences observed in *ets-4(uz1)* worms, more genes (542) displayed altered expression in these animals than in *ets-4(ok165)* worms (Table S3). qRT-PCR controls in age-matched, 1-day old *ets-4(uz1)* and wild-type adult worms confirmed the expression changes observed for eight out of eight genes (Figure S4).

Because animals carrying either *ets-4* deletion displayed similar life span extension, we predicted that the genes overlapping in these data sets would be enriched for aging effectors. A statistically significant overlap of 70 genes with altered expression in *ets-4(ok165)* and *ets-4(uz1)* worms was identified (p<0.0001) (Table 2 and Table S4). To determine, in an unbiased manner, whether these 70 ETS-4-regulated genes represented a particular biological pathway, we performed gene ontology analysis using GOstat [43]. The top five overrepresented categories yielded by this analysis include lipid transport (*vit-2, vit-3*, *vit-4*, and *vit-5*), multicellular organismal aging (*vit-5*, *vit-2*, *thn-1*, and *lys-7*) and fatty acid metabolic process (*acdh-2*, *ech-9*, and *C48B4.1*) (Table 3). Five control gene lists of the same size generated randomly from genes represented on the expression arrays did not show these classes to be overrepresented.

**Table 2:** Comparison of Genes Altered in *ets-4* Mutant Worms to Other Published Gene Lists.

Data Set-A	Data Set-B	Percent Overlap^a^	p-value^b^
*ets-4(uz1)* e	*ets-4(ok165)* e	48%	<0.0001^c^
Aging genes^g^	ETS-4-regulated^e^,^f^	24%	<0.0001^c^
*daf-16* genes^h^	ETS-4-regulated^e^, ^f^	20%	<0.0001^c^
*skn-1* genes^i^	ETS-4-regulated^e^, ^f^	0%	NS^d^
Intestine enriched genes^j^	ETS-4-regulated^e^ ^f^	14%	<0.0001^c^
Pharynx enriched genes^k^	ETS-4-regulated^e^, ^f^	1%	NS^d^
Muscle enriched genes^l^	ETS-4-regulated^e^, ^f^	0%	NS^d^
Germ-line enriched genes^m^	ETS-4-regulated^e^, ^f^	0%	NS^d^
Neuron enriched genes^n^	ETS-4-regulated^e^, ^f^	3%	NS^d^

a Percent of data set-B that overlaps with data set-A.

b p-values generated from unpaired two-tailed t-test.

c ψ^2^ analysis yielded statistically significant values for these pair-wise comparisons.

d Not significant, NS. p-values>0.05 were not listed.

e Data from this study.

f 70 genes with altered expression in *ets-4(ok165)* and *ets-4(uz1)* worms relative to wild-type worms.

g Budovskaya et al, 2008;

h Murphy et al, 2003;

i Park et al, 2009;

j Pauli et al, 2006;

k Gaudet et al, 2002;

l Roy et al, 2002;

m Reinke et al, 2000;

n Watson et al, 2008.

**Table 3:** Top Five Overrepresented Ontology Terms of ETS-4-Regulated Genes^a^.

Gene Ontology Term	Percentage^b^	p-value^c^
Lipid transport	5.7%	2.3×10^−7^
Multicellular organismal aging^d^	5.7%	2.1×10^−3^
Determination of adult lifespan^d^	5.7%	2.1×10^−3^
Acyl-CoA dehydrogenase activity	2.9%	2.3×10^−3^
Fatty acid metabolic process	2.9%	5.5×10^−3^

a 70 genes with altered expression in *ets-4(ok165)* and *ets-4(uz1)* worms relative to wild-type worms.

b Percentage of ETS-4-regulated genes that fit the gene ontology term.

**^c^**p-values were generated by GOstat (Beissbarth et al, 2004).

d These ontology terms represent the same set of genes.

Restoration of ETS-4 function in the intestine rescued life span of *ets-4* null worms to wild-type (Figure 3D and Table 1). To test whether ETS-4-regulated genes were biased towards expression in a particular cell type, we compared our microarray data set to tissue-specific expression data from previous studies. Strikingly, the ETS-4-regulated gene list was significantly (p<0.0001) enriched only with intestinal genes [44], and not with germ-line [45], muscle [46], pharyngeal [47] or neuronal genes [48] (Table 2). This result did not vary when down- or up-regulated gene sets in the *ets-4* mutant animals were analyzed separately for enrichment of cell-type specific gene expression (data not shown). Thus, the molecular signature of ETS-4, identified by gene expression profiling, substantiates a role for *ets-4* in the intestine.

Lipid storage and metabolism have been linked to longevity regulation [36], [39], [40], [49] Also, expression of yolk protein genes (*vit* genes) and genes regulating fatty acid β-oxidation, such as an acyl-CoA dehydrogenase (*acdh-2*), an acyl-CoA oxidase (*C48B4.1*) and an enoyl*-*CoA hydratase (*ech-9*), were down-regulated in *ets-4* mutant animals (Table S4). Thus, to explore a possible mechanism for life span regulation by ETS-4, we examined lipid levels in *ets-4* null mutant animals. Lipid extracts from synchronized, one-day old adult, wild-type and *ets-4* null mutant animals were subjected to thin-layer chromatography (TLC). The phospholipid (PL) and triacyglyceride (TAG) fractions from *ets-4(ok165)* worms visualized on TLC plates were similar to wild-type (Figure S5). The relative levels of triacylglycerol stores, as well as the fatty acid composition of phospholipid and triacylglycerol fractions, quantified by gas chromatography in age-matched, one-day old *ets-4(ok165)* adult animals, were not altered compared to wild-type worms (Figure S5). Thus, the altered expression of genes involved in lipid metabolism did not affect total lipid levels in *ets-4(ok165)* worms. Therefore, lipid homeostasis in *ets-4(ok165)* animals could likely be maintained due to the compensatory activities of other enzymes regulating fatty acid metabolism. Alternatively, lipid uptake, transport or storage in different tissues of *ets-4(ok165)* worms may be affected without an alteration in the total lipid levels within the whole organism.

Discerning the transcriptional targets of ETS-4 enabled us to ask whether ETS-4-regulated genes change expression during the course of normal aging [3]. We found that 24% of the 70 ETS-4-regulated genes were previously identified age-regulated genes (Table 2 and Table S4). This is a significantly higher overlap than that expected by random chance (p<0.0001), corroborating a function for ETS-4 in normal aging (Table 2). Interestingly, the GATA transcription factors ELT-3, ELT-5, and ELT-6 were shown to direct the age-regulation of a large fraction of genes that change expression with age [3]. Our data suggested that ETS-4 participated in directing the expression of a significant proportion of age-regulated genes.

To decipher whether a common set of targets exists for transcription factors that function in life span determination, we compared ETS-4-regulated genes with those that function downstream of the well-characterized insulin/IGF-1 signaling pathway. A comparison to the genes that act downstream of two components of the insulin/IGF-1 signaling pathway (*daf-2* and *daf-16*) [10] indicated a 20% overlap, which represents a significant enrichment (p<0.0001) (Table 2 and Table S4). The overlapping gene set included genes that were down-regulated as well as genes up-regulated in *ets-4(ok165)* animals (Table S4). Also, 50% of the overlapping genes were up-regulated in *daf-2* pathway mutant animals and repressed in *daf-16; daf-2* double mutant animals, while the other half displayed the opposite expression profile (Table S4) [10] Since *ets-4* and *daf-2* loss-of-function mutations cause an extended life span phenotype, we predicted that the direction of expression changes for genes involved in aging will be similar in these mutant animals. Indeed, the genes identified by our ontology analyses as functioning in multicellular organismal aging (Table 3) were either down-regulated (*vit-5* and *vit-2*) or up-regulated (*thn-1* and *lys-7*) in both *ets-4(ok165)* and *daf-2(−)* animals (Table S4) [10]. Taken together, our analyses revealed a shared pattern of gene expression changes between in *ets-4* mutant animals, insulin/IGF-1 signaling pathway mutant animals, and aging wild-type worms. Thus, a common set of transcriptional targets for ETS-4, the GATA factors (ELT-3, ELT-5, and ELT-6), and DAF-16 exist.

### ETS-4 Functions as a Sequence-specific DNA Binding Transcription Factor

**Figure 5:**
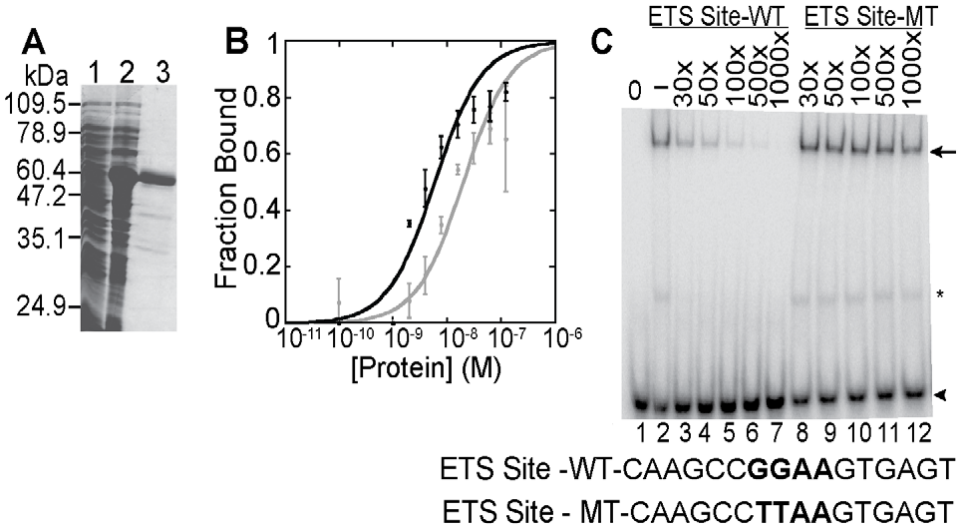
*In vitro* DNA binding activity of ETS-4. (A) Purification of recombinant ETS-4. SDS-PAGE gel, stained with coomassie blue, shows uninduced (lane 1), induced (lane 2) bacterial lysate and purified 6xHis tagged ETS-4 (lane 3). (B) DNA-binding isotherms derived from electrophoretic mobility shift assays (EMSAs) performed with increasing concentrations of recombinant ETS-4 (10^−11^ M to 10^−7^ M) incubated with radiolabeled DNA duplex containing a GGAA (black)- or GGAT (gray)-based ETS binding site. Binding curves represent the mean (±SD) from three independent EMSA experiments. (C) EMSA showing sequence specificity of ETS-4 binding. Radiolabeled DNA duplex (ETS Site-WT) was used at 10^−10^ M for all lanes. Unlabeled competitor DNA duplex was added in molar excess (X) (lanes 3–12). The competitor DNA contained either a GGAA sequence (ETS Site-WT) or a mutated sequence (TTAA) (ETS Site-MT). The arrowhead marks the mobility of unbound DNA. Binding of ETS-4 shifts the DNA to a slower migrating band (arrow). Band marked by asterisk is possibly due to an ETS-4 degradation product bound specifically to DNA.

Analyzing global transcriptional changes in *ets-4* mutant animals identified a number of genes regulated by ETS-4. Direct transcriptional targets are predicted to have ETS-4 binding sites in the transcription start site (TSS)-proximal regions. As a first step towards testing this hypothesis, we characterized the DNA binding and transcriptional activity of ETS-4. We tested the binding of ETS-4 to ETS binding sites previously described, which display a 5′-GGAA/T-3′ core recognition sequence [13], [50], which is consistent with the reported motif for the vertebrate orthologue, SPDEF [51]. ETS-4, purified from a bacterial expression system, bound to ETS binding sites displaying either a GGAA or GGAT core motif with similar high affinity (K_D_∼10^-9^ M) (Figure 5A and Figure 5B). Furthermore, a wild-type, but not mutated, ETS binding site, competed with ETS-4 binding to the labeled DNA (Figure 5C). These results indicated that ETS-4 bound to consensus ETS sites in a sequence-specific manner.

ETS proteins demonstrate functional diversity acting both as transcriptional activators and repressors [13],[52]. To determine the transcriptional activity of ETS-4, we tested ETS-4 in transcription assays in yeast and cultured mouse fibroblasts. In *S. cerevisiae*, LexA::ETS-4(1-345) activated transcription of a reporter gene via a promoter with LexA binding sites (Figure S6). ETS-4(1–345) also activated transcription in NIH3T3 cells (Figure S6). In yeast, the N-terminal ETS-4(1–125) fragment activated transcription of the reporter gene; however, this activity was lost with the inclusion of the PNT domain in ETS-4(1–200) (Figure S6). Indeed, we have mapped repressive function to the PNT domain including recruitment of repressive co-factors (data not shown). Thus, our data identify two regions of ETS-4 that activate transcription (amino acids 1–125 and 200–345) and suggest the ability of the PNT domain to modulate this activity towards a repressive function. This potential opposing transcriptional function is consistent with the hypothesis that both up- and down-regulated genes are direct ETS-4 targets.

To identify candidate direct targets of ETS-4, we looked for ETS binding sites conserved across six nematode species in the region from 1500 base pairs (bp) upstream to 500 bp downstream of the transcription start site (TSS) of ETS-4-regulated genes. The position specific weight matrix (PWM) used to search for ETS binding motifs encompasses the sites that ETS-4 bound *in vitro* (Figure 5 and Table S5). Using the MULTIZ alignment algorithm [53], at least one conserved ETS binding motif was identified in the transcriptional control regions of 54 of the 70 ETS-4 regulated genes (Table S5). Although this frequency did not represent an enrichment of conserved ETS sites compared to other randomly selected TSS-proximal regions (data not shown), we suspect this represents the general importance of ETS factors in many control regions and not the lack of biological relevance of these sites on ETS-4 regulated genes [54], [55]. It was striking that genes with conserved ETS binding motifs included not only 82% of the down-regulated genes, but also 72% of the up-regulated genes in *ets-4* mutant animals (Table S5). This is consistent with the activating and repressive transcriptional functions of ETS-4 noted above. Also, several ETS proteins, in response to signaling pathways, fine-tune their transcriptional activity, functioning as activators or repressors [13], [52], [56]. In conclusion, we propose that ETS proteins may bind the transcriptional control regions and, thus, regulate directly the expression of 77% of the genes that were identified to function downstream of ETS-4.

To test the function of ETS-4 as a DNA binding transcription factor in a native context, we focused on *vit-5*, which is one of the genes down-regulated in *ets-4* mutant worms that bears conserved ETS binding sites (Table S2, Table S4 and Table S5). *vit-5* encodes a lipoprotein related to mammalian ApoB-100, a core LDL particle constituent [57]. Also, *vit-5(RNAi)* was shown previously to extend the mean life span of RNAi-sensitive wild-type worms [10]. *Pvit-5::gfp* expression was significantly reduced in the intestinal cells of *ets-4(ok165)* compared to wild-type worms suggesting that ETS-4 is necessary for inducing *vit-5* expression (Figure S7). We conclude that ETS-4 is a transcription factor, fully competent to help orchestrate a transcriptional network involved in life span regulation.

### ETS-4 Functions in Parallel to the Insulin/IGF-1 Receptor Signaling

**Figure 6:**
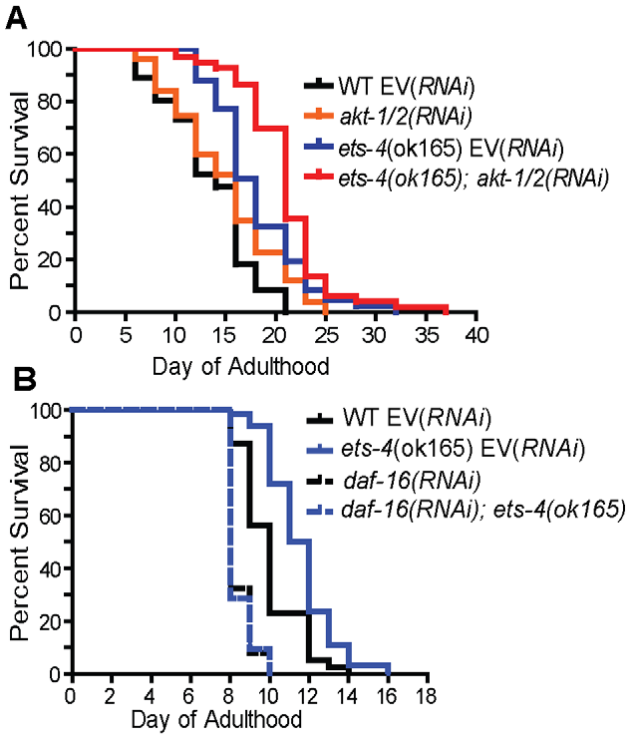
*ets-4* acts in parallel to the insulin/IGF-1 signaling pathway converging onto *daf-16*. Survival curves from life span experiments conducted at 25°C. See Table 1 and Table S1 for data from additional trials, mean life span and statistical analysis. (A) Wild-type (WT) and *ets-4(ok165)* worms were grown at 25°C and subjected to *akt-1/2(RNAi)* or an empty vector (EV) control RNAi starting at the L4 stage. (B) Wild-type (WT) and *ets-4(ok165)* worms were grown at 25°C and subjected to *daf-16(RNAi)* or an empty vector (EV) control RNAi starting at the L4 stage.

**Figure 7:**
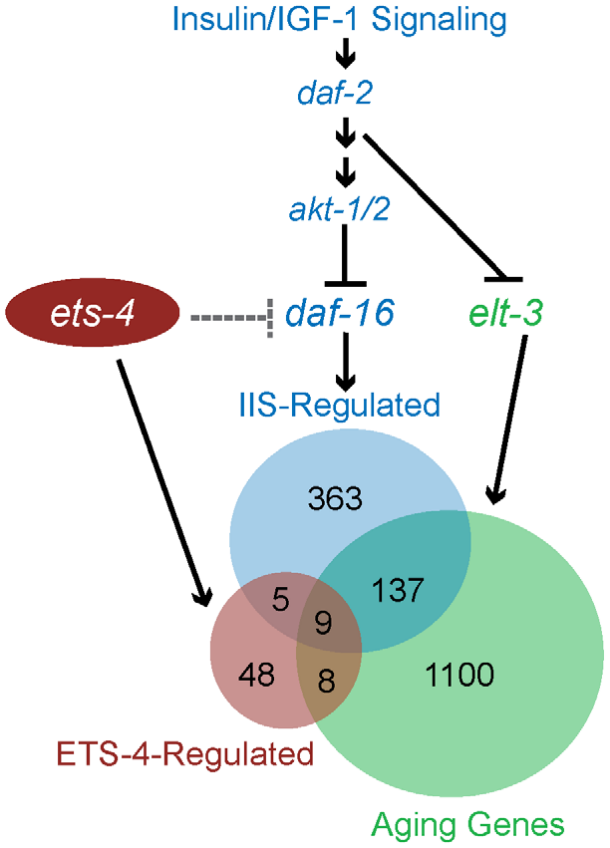
Model for ETS-4 function as a transcriptional regulator. ETS-4 functions in parallel to the insulin/IGF-1 signaling pathway (blue) to modulate a set of downstream targets (red circle). *ets-4* may function upstream of (dotted line) or in parallel (solid line) to antagonize *daf-16* function in longevity regulation. ETS-4-regulated gene set overlaps with that of the insulin/IGF-1 signaling pathway acting through DAF-16 (blue circle) as well as that of ELT-3 (green circle), which directs the age-regulation of a majority of genes that change expression with age.

To characterize how *ets-4* modulates *C. elegans* lifespan, we tested whether *ets-4* genetically interacted with other known longevity regulators. First, because ETS-4, ELT-3, and DAF-16 share common downstream targets, we asked if they function downstream of the same signaling pathway. The insulin/IGF-1 signaling pathway involves the activation of the insulin/IGF-1 receptor, DAF-2, which triggers a kinase cascade involving the serine/threonine kinases AKT-1 and AKT-2 culminating in the cytoplasmic sequestration and inhibition of the FOXO transcription factor DAF-16 [58], [59]. In addition to inhibiting DAF-16, the insulin/IGF-1 pathway also exerts a constant level of regulation on ELT-3 expression [3], [60]. We tested whether *ets-4* modulated life span by acting downstream of the insulin/IGF-1 signaling pathway. To do this, we examined the genetic relationship between *ets-4* and two major components of the insulin/IGF-1 signaling cascade - the receptor *daf-2* and the kinases *akt-1/2*. For the life span comparisons, RNAi was used to inactivate insulin/IGF-1 signaling pathway genes in isogenic *ets-4(ok165)* and wild-type strains. If *ets-4* regulates life span by acting downstream of the insulin/IGF-1 signaling pathway, then loss of *ets-4* should not significantly extend the life span of long-lived worms lacking insulin/IGF-1 signaling. Alternatively, further extension of the life span of long-lived *ets-4* null mutant worms upon inhibition of insulin/IGF-1 signaling would indicate that *ets-4* functions in parallel to this pathway. As previously reported [1], [61], [62], *daf-2(RNAi)* animals were significantly long-lived (Table 1). Notably, *daf-2(RNAi); ets-4(ok165)* animals lived longer than either *ets-4(ok165)* or *daf-2(RNAi)* worms alone (Table 1 and Table S1). Similarly, RNAi against the kinases *akt-1/akt-2* further extended the life span of the long-lived *ets-4(ok165)* worms (Figure 6A and Table 1). Thus, inhibition of two different components of the insulin/IGF-1 signaling pathway further increased the life span of long-lived *ets-4(ok165)* animals. Because RNAi, and not null mutations, was used to inactivate signaling pathway genes, the possibility that the insulin/IGF-1 receptor pathway partially contributes to the life span phenotypes of *ets-4* null mutant animals cannot be completely eliminated. However, our data support a model whereby *ets-4* functions in parallel to the insulin/IGF-1 signaling pathway to modulate life span (Figure 7).

We further explored the genetic relationship between *ets-4* and the insulin/IGF-1 signaling pathway by investigating if *ets-4* participated in other physiological processes regulated by the pathway. Signaling through DAF-2 is critical for dauer formation. Hence, the involvement of *ets-4* in dauer formation was tested first. Assaying for dauer formation at 25°C and 27°C [63], [64], we found no defects in dauer formation in *ets-4(ok165)* mutant animals compared to wild-type worms (data not shown), suggesting that *ets-4* does not play a significant role in this process. Next, we tested whether *ets-4* null mutant animals exhibit altered response to environmental stress stimuli since insulin/IGF-1 signaling also regulates stress resistance. To assay for heat stress response, the survival of adult *ets-4(ok165)* worms was monitored after a shift to 35°C. As reported previously[65]-[68], *daf-2(e1370)* animals survived significantly longer than wild-type worms at 35°C, whereas *daf-16(mgDf50)* animals died faster (Figure S8). No significant differences in survival were seen between *ets-4(ok165)* and wild-type worms during the heat stress time-course (Figure S8), indicating that *ets-4* is not required for response to heat stress.

Extension of life span is often associated with increased resistance to oxidative stress [69]. We determined whether *ets-4* null mutant animals show altered response to oxidative stress by monitoring survival when exposed to a powerful oxidant, paraquat. No significant differences in survival were seen between *ets-4(ok165)* and wild-type worms during the majority of the oxidative stress time-course (Figure S8), indicating that *ets-4(ok165)* worms display wild-type response to oxidative stress. The participation of ETS-4 in the oxidative stress response pathway was also tested by assessing the genetic interaction of *ets-4* with *skn-1*. Transcription factor SKN-1, the ortholog of mammalian Nrf proteins, is critical for oxidative stress resistance and acts in multiple longevity pathways, including the insulin/IGF-1 signaling cascade [70]. To test whether SKN-1 was required to mediate the life span extension observed in *ets-4* null mutant animals, we inhibited *skn-1* activity by RNAi and monitored the adult life span at 25°C. *skn-1(RNAi)* was previously reported to alter the expression of genes involved in oxidative stress response [71]. Also, inhibition of *skn-1* by RNAi was shown to decrease the life span of *daf-2* null mutant animals, but not control RNAi sensitive animals [72]. *skn-1(RNAi)* on *ets-4(ok165)* worms did not alter the extended life span phenotype of these worms (Figure S8), suggesting that the *ets-4* null mutant animals are not susceptible to a partial loss of SKN-1 function. Additionally, to determine whether ETS-4 and SKN-1 shared downstream effectors, we compared a list of SKN-1-dependent target genes involved in oxidative stress response [71] to the 70 ETS-4-regulated genes. There was no significant enrichment for the stress responsive, SKN-1-dependent genes amongst genes that act downstream of ETS-4 (Table 2). Taken together, these data suggest that ETS-4 does not contribute to all physiological processes regulated by insulin/IGF-1 signaling and supports a model whereby ETS-4 functions, in part, independently of DAF-2 signaling to regulate life span.

### ETS-4 Requires DAF-16 to Regulate Life Span

The FOXO transcription factor, DAF-16, is a well established regulator of life span that functions in the insulin/IGF-1 signaling pathway [1], [60], [73]–[75] and shares downstream longevity effectors with ETS-4 (Table 2 and Figure 7). We examined the genetic relationship between *ets-4* and *daf-16*. Consistent with previous studies [1], [61], [75], inhibition of *daf-16* activity by RNAi shortened life span (Figure 6B and Table 1). Further, *ets-4(ok165)* worms, when subjected to *daf-16(*RNAi*)*, did not display an extended life span (Figure 6B and Table 1). Thus, *daf-16* was required for the longevity phenotype of *ets-4(ok165)* worms suggesting that *ets-4* functions upstream of, or in parallel to, *daf-16* in lifespan regulation (Figure 7). Because the loss of *ets-4* extends life span, whereas, inhibition of *daf-16* activity shortens it, our genetic tests indicate that *ets-4* antagonizes *daf-16* function in longevity regulation by upstream effects, or in parallel.

We examined in more detail two possible ways by which *ets-4* could function upstream of *daf-16*. First, we tested whether ETS-4 reduced DAF-16 levels through a transcriptional effect. Our gene expression profiling experiments indicated that the expression of *daf-16* transcripts was not altered in *ets-4* mutant worms (NCBI's Gene Expression Omnibus, accession number GSE17954). Further, upon crossing a *daf-16::gfp* reporter into *ets-4(ok165)* worms, we did not observe an alteration in the expression of DAF-16::GFP due to loss of *ets-4* (data not shown). Second, we examined whether ETS-4 antagonized DAF-16 activity by promoting its cytoplasmic retention. The function of the transcription factor DAF-16 is modulated in response to stress conditions, such as heat-shock, by altering its nuclear localization [58], [76]. The intracellular localization of DAF-16::GFP was not affected by the loss of *ets-4* under normal growth conditions and heat-shock (data not shown). These results suggest that ETS-4 does not regulate DAF-16 by altering its expression level or nuclear-cytoplasmic localization. Alternatively, ETS-4 and DAF-16 could function in parallel pathways to modulate distinct targets involved in longevity regulation (Figure 7). Or, an indirect modulation of DAF-16 activity by ETS-4 is possible, through the regulation of a required transcription co-factor. Taken together, we conclude that ETS-4 is a new life span determinant that functions in parallel to the insulin/IGF-1 signaling pathway, but requires the FOXO transcription factor, *daf-16*, to modulate life span.

## Discussion

In this study, we describe a novel transcriptional regulator of longevity. Worms lacking ETS-4 exhibited a pronounced life span extension. Despite broad expression in multiple cell types, ETS-4 function in the adult intestine was important for longevity regulation. Notably, identification of an ETS-4-regulated gene set uncovered shared transcriptional targets with two other longevity modifiers, the FOXO and GATA factors.

### ETS-4 Regulates Life Span

Loss of *ets-4* function led to a substantial extension in mean adult life span. Additionally, *ets-4* mutant worms exhibited an altered egg-laying rate. These data are consistent with the precedence of several life span altering mutations that affect multiple aspects of nematode biology, including reproduction and metabolism. This raises the question of whether these phenotypes are causative of increased longevity. Another key question with the identification of ETS-4 as a novel genetic modifier of aging with a broad expression pattern was whether a particular cell type was crucial for its function in longevity regulation. Our data showed that restoring ETS-4 function specifically in the intestine, but not neurons, rescued the extended life span of *ets-4* null mutant animals back down to wild-type levels. Interestingly, other transcription factors that function as longevity determinants, including the FOXO protein DAF-16 and the GATA factors ELT-3, ELT-5, and ELT-6, regulate life span primarily through their function in the intestine [3], [34]. These data thus also support our model of shared transcriptional targets for these factors. Moreover, restoring expression of ETS-4 in the intestine of null mutant worms does not rescue the altered egg-laying phenotype. Thus, the longevity and altered egg-laying rate phenotypes are separable, implying a correlative rather than causative relationship between the two. Because relative to one another, the rates of aging of different tissues appear normal, it is proposed that a network of signaling and feedback regulation is involved in coordinating aging within an animal [33], [34]. Our data demonstrate that ETS-4 is a transcriptional regulator of this network that functions in the intestine to modulate the rate of aging in *C. elegans*.

### Model for ETS-4 Function in Longevity Regulation

Gene expression profiling of long-lived worms carrying two different deletion alleles of *ets-4*, identified a robust set of ETS-4-regulated genes. Consistent with the longevity phenotype of *ets-4* mutant worms, the ETS-4-regulated gene set was enriched for genes that modulate life span (Table 3). In addition to the regulation of these known life span determinants by ETS-4, 29 genes of unknown function were also misexpressed in *ets-4* mutant animals and may contribute to the long-lived phenotype of these worms. DNA-binding studies and bioinformatics searches identified conserved ETS binding sites in promoter regions of 54 of the 70 ETS-4-regulated genes, suggesting that these were direct targets (Table S5). Notably, the ETS-4-regulated gene set was significantly enriched for genes that change expression during normal aging (Figure 7, Table 2 and Table S4), the majority of which were proposed targets of the GATA factor ELT-3 [3]. Consistent with the shared pattern of expression changes observed between aged worms and insulin/IGF-1 signaling pathway mutant animals [3], a significant proportion of ETS-4-regulated genes functioned downstream of the insulin/IGF-1 signaling pathway and the FOXO transcription factor DAF-16 [10], [11] (Figure 7, Table 2, and Table S4). RNAi against four of these common downstream effectors, *vit-5*, *vit-2*, *thn-1*, *lys-7* alters worm life span [10]. These longevity effectors participate in lipid transport (vitellogenins/yolk proteins) and innate immune response (lysozymes and thaumatins). In addition to this set of overlapping targets for three transcriptional regulators of aging ELT-3, DAF-16 and ETS-4, notable targets were absent. For example, expression of genes mediating response to oxidative stress regulated by the transcription factor SKN-1 (superoxide dismutases) [71], heat-shock or toxicity (metallothioneins and xenobiotic metabolism genes) that are altered in *daf-16* mutant worms remain unchanged in long-lived *ets-4* mutant animals. We propose that ETS-4 participates in some, but not all biological processes regulated by FOXO and GATA factors. In summary, we introduce ETS-4 as a novel transcriptional regulator in the genetic network that modulates life span.

### Insights into ETS function in Vertebrate Development and Disease

Our study is the first demonstration of an ETS factor modulating animal life span, thus illustrating the utility of *C. elegans* in identifying novel functions for ETS proteins not easily discerned in more complex systems. The mammalian ortholog of *ets-4*, *SPDEF* is expressed in the intestine and in tissues with high epithelial content, like breast and prostate [17], [77]. A recent study of a mouse mutant strain with a dysfunctional *SPDEF* allele showed impaired terminal differentiation of specialized intestinal secretory cells derived from the intestinal epithelium [24]. Markers for these secretory intestinal cells, the Paneth and goblet cells, were implicated as SPDEF targets [24]. In an interesting evolutionary convergence, our study illustrated ETS-4 function in the worm intestine, which is a tube comprised of 20 large epithelial cells [78]. The *C. elegans* intestine executes multiple functions carried out by distinct organs in higher eukaryotes, such as digestion and absorption of nutrients, synthesis and storage of fats, initiation of an innate immune response to pathogens and yolk production [44], [78]–[80]. Our work demonstrates the regulation of intestinal genes, such as lysozymes and vitellogenins by ETS-4. Additionally, restoring ETS-4 function in the worm intestine, but not neurons, reduced the life span of *ets-4* null worms to wild-type, revealing a link between ETS function in the intestine and longevity regulation.

Our finding that ETS-4 has a role in *C. elegans* aging also provides a new connection between this ETS factor and cancer. Studies in human cell lines have implicated SPDEF function in tumorigenesis. Cell lines from breast and prostate tumors have altered SPDEF expression, although the significance of this misregulation remains controversial. Whereas some studies propose a putative role for SPDEF as a tumor suppressor, others suggest a prometastatic function [17], [18], [22], [23], [81], [82]. Given the strong correlation between physiological aging and tumor susceptibility, aging studies in *C. elegans* have been used to provide genetic insights into tumor biology [83]–[85]. Thus, the role of ETS-4 in aging raises new implications for the physiological role of mammalian ETS factors in development, homeostasis and disease.

## Materials and Methods

### *C. elegans* Strains and RNAi

*C. elegans* strains were maintained at 20°C as described previously [86] unless otherwise mentioned. The wild-type reference strain was N2 Bristol. The RB637: *ets-4(ok165)* X strain was obtained from the *C. elegans* Gene Knockout Project at OMRF (International *C. elegans* Gene Knockout Consortium). *ets-4(uz1)* was isolated by PCR-based screening of a library of worms mutated with EMS generated at the University of Utah. The deletion breakpoints of the *uz1* on cosmid F22A3 are 14790 and 15861. Single-worm PCR [87] was used for screening and to determine the genotype of worms during crosses with *ets-4(uz1)* and *ets-4(ok165)* worms. For *ets-4(ok165)* and *ets-4(uz1)* identification, nested-PCR was carried out using the following primers: Forward primers: CAATGAACGGTACTGGCTCAG and Primer P5: TGCAATCTTCCAATCCAACCC; Reverse primers: ACTGCCGGAGGACAAATGTC and Primer P6: CATTGCGATTCCCATGTAACC; Primers for sequences deleted in the *ok165* and *uz1* alleles: GCTAGCCAGCACCAACAATCAA and ACACCAAACGCTGCTTCTTT. The *ets-4(ok165)* and *ets-4(uz1)* worms were outcrossed to the N2 strain six times before phenotypic analysis, including life span assays. We also used BC11290: dpy-5(e907) I; sEx11290[rCesC04F6.1::GFP + pCeh361] [31], TJ356: zIS356 IV [Pdaf-16::daf-16-gfp; rol-6(su1006)] [76], *lin-15(n765ts)* [88], *daf-16(mgDf50) I*, CF1041 *daf-2(e1370)* III [66].

RNAi was performed essentially as described previously [89] using HT115(DE3) *E. coli* expressing dsRNA from *daf-16*, *daf-2*, *skn-1* (obtained from Julie Ahringer's RNAi library [90]), *akt-1*, *akt-2* (obtained from the Marc Vidal's RNAi library [91]), *ets-4* or carrying the L4440 control plasmid (EV for empty vector control). Each clone was sequenced to confirm its identity. Individual RNAi clones were grown overnight with Ampicillin (100 µg/ml) or Kanamycin (25 µg/ml) seeded onto NGM plates containing 1 mM IPTG (Sigma) and 25 µg/ml Carbenicillin (Sigma) and allowed to grow for 2 days at room temperature. For *akt-1/2(RNAi)*, a 1:1 mix of *akt-1* and *akt-2* equal density overnight bacterial cultures was used to seed NGM plates containing 1 mM IPTG (Sigma) and 25 µg/ml Carbenicillin (Sigma). L4 stage larvae were placed on the RNAi plates and observed for phenotypes at 25°C.

### RT-PCR and Microarray Analysis

Total RNA from mixed stage worms was isolated by phenol-chloroform extraction and subjected to DNaseI treatment using the RNeasy kit (Qiagen). 1–2 µg of total RNA was reverse-transcribed by SuperScript III (Invitrogen) according to manufacturer's protocol. Ten percent of the resultant cDNA was PCR-amplified by *Taq* DNA polymerase in a 50 µl reaction. Primer sequences: R1: GGCACAAGTTGTACTGATGTC, P1 (SL1 primer): GTTTAATTACCCAAG TTTGAG, P2: CAGATGACGGAGAATCAGGTC, R2: CTACAAGTTATAAGGAGGCAGG, P3: CTTCAGCCGCCTAGAAACTG and P4: CCAATATCTAGCCAGCAGGAG. The PCR product was sequenced to determine the position of SL1 attachment and splicing pattern.

To assess the expression of mRNA in synchronized populations of N2, *ets-4(ok165)* and *ets-4(uz1)* worms, levels of cDNA from the reverse transcription reaction were assessed by quantitative PCR according to manufacturer's protocol using the Roche LightCycler 480. Transcript levels were normalized to the averaged levels of *cdc-42* and *pmp-3* [92]. See Table S6 for primer sequences.

For microarray analysis, total RNA was isolated from L4 stage larvae as described above and reverse transcribed. Cy5 and Cy3 labeled cDNA was applied to *C. elegans* 22K gene expression arrays (Agilent). Data from three independent repeats of the experiment were analyzed. Lowess-normalized log (base 10) ratios of mutant/wild-type gene expression were obtained from the two-color Agilent *C. elegans* gene expression microarrays. The log ratios were analyzed using the Rank Products method [93] to identify consistently differentially expressed genes. Genes were selected using a probability of false prediction (PFP) cut off of 0.1, i.e. a false discovery rate of 10%. This technique corrects for multiple testing with repeated trials on random permutations of the data set.

The microarray data discussed in this publication have been deposited in NCBI's Gene Expression Omnibus (Edgar *et al*., 2002) and are accessible through GEO Series accession number GSE21851 (http://www.ncbi.nlm.nih.gov/geo/query/acc.cgi?acc=GSE21851).

### Life Span Assays

Life span assays were conducted as described previously [94]. The *ets-4(ok165)* and *ets-4(uz1)* worms were outcrossed to the wild-type strain six times before the life span assays. Briefly, worms were grown for two or more generations at the assay temperature (25°C or 20°C) prior to the assay. Hermaphrodites were allowed to lay eggs for 6–8 hr on OP50 to obtain synchronous progeny for the experiment. L4 stage larvae were picked to plates spotted with RNAi bacteria containing 1 mM IPTG or OP50 bacteria and allowed to age at 25°C. The animals were moved to fresh plates daily during the reproductive period and every other day for the rest of the assay. The worms were scored for life by assessing movement to touch every 1–2 days [8]. Animals that bagged, exploded or crawled off the plate were excluded from the analysis. The first day of adulthood was counted as day one of the life span experiment. Life span curves and statistical data including p-values from Log-rank (Mantel-Cox) test were generated using GraphPad Prism version 5 software (GraphPad Software, San Diego, California USA).

### Brood Size, Egg-Laying Rate and Development Time Assays

Single L4 stage larvae were allowed to lay eggs at 20°C and transferred to a fresh NGM plate every day till the end of the reproductive period. The number of eggs laid and the number of hatched progeny were counted. The average number of eggs laid each day during the egg-laying period and the total number of progeny per worm (brood size) were plotted. Development time assays were done as described previously [95]. Briefly, synchronized L1 larvae of each genotype were grown at 20°C. The animals were monitored every 3 hr after they had reached L4 stage till they were pre-fertile adults. Each experiment with at least ten worms per genotype was repeated twice, independently. Unpaired t-test analyses were performed to calculate p-values.

## Supporting Information

Figure S1 Phylogenetic analysis of ETS domain sequences. The protein sequences of 27 *H. sapiens* (gray) and 10 *C. elegans* ETS domains (Ce) were aligned using ClustalW (version 1.83). DRAWGRAM (from phylip version 3.66) was used to construct a dendrogram of the aligned sequences. The horizontal length of the branches predicts the evolutionary distance between the genes.
(9.87 MB TIF)

Figure S2 *ets-4* mutations do not alter feeding rate or defecation rate. (A) Pharyngeal pumping rates (pumps/minute) were counted for wild-type (WT) (n = 11), *ets-4(ok165)* (n = 10) and *ets-4(uz1)* (n = 12) worms (mean ± SEM). (B) Mean defecation cycle periods (seconds/cycle) of wild-type (WT), *ets-4(ok165)* and *ets-4(uz1)* worms (mean ± SEM). At least 10 worms per genotype were observed for 10 cycles each.
(3.76 MB TIF)

Figure S3 Tissue-specific expression of ETS-4 does not affect the egg-laying rate or brood size of *ets-4(ok165)* animals. (A) The number of eggs laid (mean ± SEM) during each day of the egg-laying period. The egg-laying rate of wild-type (WT) worms, *ets-4(ok165)* Control and that of strains with tissue-specific expression of *ets-4* in intestinal cells, *ets-4(ok165);[Pgly-19::yfp::ets-4]*, or neurons, *ets-4(ok165);[Prab-3::yfp::ets-4]* at 20°C. * indicates p<0.001, comparing *ets-4(ok165)* transgenic lines to WT. (B) Total number of progeny (brood size) was counted for the genotypes indicated at 20°C. The average brood size (mean ± SEM) of *ets-4(ok165)* Control, *ets-4(ok165);[Pgly-19::yfp::ets-4]* and *ets-4(ok165);[Prab-3::yfp::ets-4]* are indicated.
(7.80 MB TIF)

Figure S4 Expression levels of selected genes in *ets-4(ok165)*, *ets-4(uz1)* and wild-type worms. Gene expression quantified by real-time PCR in *ets-4(ok165)* and *ets-4(uz1)* mutant worms relative to wild-type (WT). Error bars represent standard error. (A) Expression of genes significantly down-regulated in *ets-4(ok165)* worms (shown are *vit-2*, *vit-3*, *vit-4*, *vit-5*, *ceh-60*, *ech-9* and *fat-7*) and *ets-4(uz1)* worms (shown are *vit-2*, *vit-3*, *vit-4*, *vit-5*, *ceh-60* and *ech-9)* relative to WT. (B) Expression of genes significantly up-regulated in *ets-4(ok165)* and *ets-4(uz1)* worms (shown are *lys-7* and *thn-1*) relative to WT.
(5.96 MB TIF)

Figure S5 Lipid levels and fatty acid composition is unaltered in *ets-4(ok165)* worms. (A) Relative abundance of triacylglycerides and phospholipids expressed as percentage of total fatty acids (±standard error) as determined by gas chromatography. (B) Relative abundance of selected fatty acid species expressed as percentage of total fatty acids (± standard error) of WT and *ets-4(ok165)* worms as determined by gas chromatography. (C) TLC analysis of lipids extracted from wild-type (WT) (lane 1) and *ets-4(ok165)* animals (replicates in lanes 2 and 3). TAG, triacylglycerides; PL, phospholipids.
(9.58 MB TIF)

Figure S6 Transcriptional activity of ETS-4. (A) Transcriptional activity of ETS-4 in *S. cerevisiae*. An yeast strain that contains an integrated LacZ reporter with 8 LexA binding sites was transformed with the indicated LexA::ETS-4 fusions. Cells lysates were subjected to a colorimetric assay to assess β-galactosidase activity (LacZ activity). The relative LacZ activity was calculated by normalizing the LacZ values to that of control strains transformed with the LexA DNA-binding domain. The expression levels of the LexA proteins were comparable (data not shown). Inset is a schematic of ETS-4. (B) Transient expression assays performed in NIH3T3 cells. Luciferase activity was measured from cells transfected with a GAL4-dependent luciferase reporter and expression vectors for GAL4 DNA-binding domain alone or as a fusion to ETS-4. Relative luciferase activity (RLA) was calculated as the ratio of firefly luciferase activity to Renilla luciferase activity (mean± SEM). The expression levels of the GAL4DBD proteins were comparable (data not shown).
(4.44 MB TIF)

Figure S7 Reduced expression of *Pvit-5::gfp in ets-4(ok165)* worms. (A) Quantification of *Pvit-5::gfp* expression in synchronized 1-day old wild-type (WT) and *ets-4(ok165)* adult hermaphrodites using ImageJ (version 1.36b) software. The mean fluorescence (±SEM) of *ets-4(ok165)[Pvit-5::gfp]* worms was measured in a fixed area at the start of the intestine and compared to that of *Pvit-5::gfp* worms in an identical area. (B) Representative example of the reduced *Pvit-5::gfp* expression in *ets-4(ok165)* worms compared to wild-type (WT) worms.
(4.60 MB TIF)

Figure S8 Stress resistance of *ets-4(ok165)* animals and genetic interaction between *ets-4* and *skn-1* for life span. (A) Survival kinetics of animals under heat stress conditions (35°C). Fraction of animals alive at different time points (hours) after a shift to 35°C (mean ± SEM) is plotted. * indicates p<0.05, compared to WT. (B) Survival kinetics of animals in the presence of paraquat (oxidative stress conditions - See Text S1). Fraction of animals alive (mean ± SEM) is plotted. * indicates p<0.05, compared to WT. (C) Survival curves for wild-type (WT) and *ets-4(ok165)* worms grown at 25°C and subjected to *skn-1(RNAi)* or an empty vector (EV) control RNAi starting at the L4 stage. See Table S1 for mean life span, statistical analyses and data from additional trials.
(10.00 MB TIF)

Table S1 Summary of Data from Repeat Trials of Life Span Analysis for *ets-4(ok165)* and *ets-4(uz1)* Mutant Worms.
(0.11 MB DOC)

Table S2 Genes with Altered Expression in *ets-4(ok165)* Compared to Wild-type (WT) Worms.
(0.09 MB DOC)

Table S3 Genes with Altered Expression in *ets-4(uz1)* compared to Wild-type (WT) Worms.
(0.26 MB DOC)

Table S4 Genes with Altered Expression in *ets-4(ok165)* and *ets-4(uz1)* Worms Relative to Wild-type Worms.
(0.10 MB DOC)

Table S5 Position of Conserved ETS Binding Motifs in the Transcriptional Control Regions of ETS-4-Regulated Genes.
(0.17 MB DOC)

Table S6 Oligonucleotide primers used in RT-PCR analyses.
(0.04 MB DOC)

Text S1 Supplemental Materials and Methods.
(0.07 MB DOC)
