# Supplementary figures and images for: ETS-4 Is a Transcriptional Regulator of Life Span in Caenorhabditis elegans
Source: PLoS Genet. 2010 Sep 16;6(9):e1001125. doi: 10.1371/journal.pgen.1001125 (PMC2940738; doi:10.1371/journal.pgen.1001125)

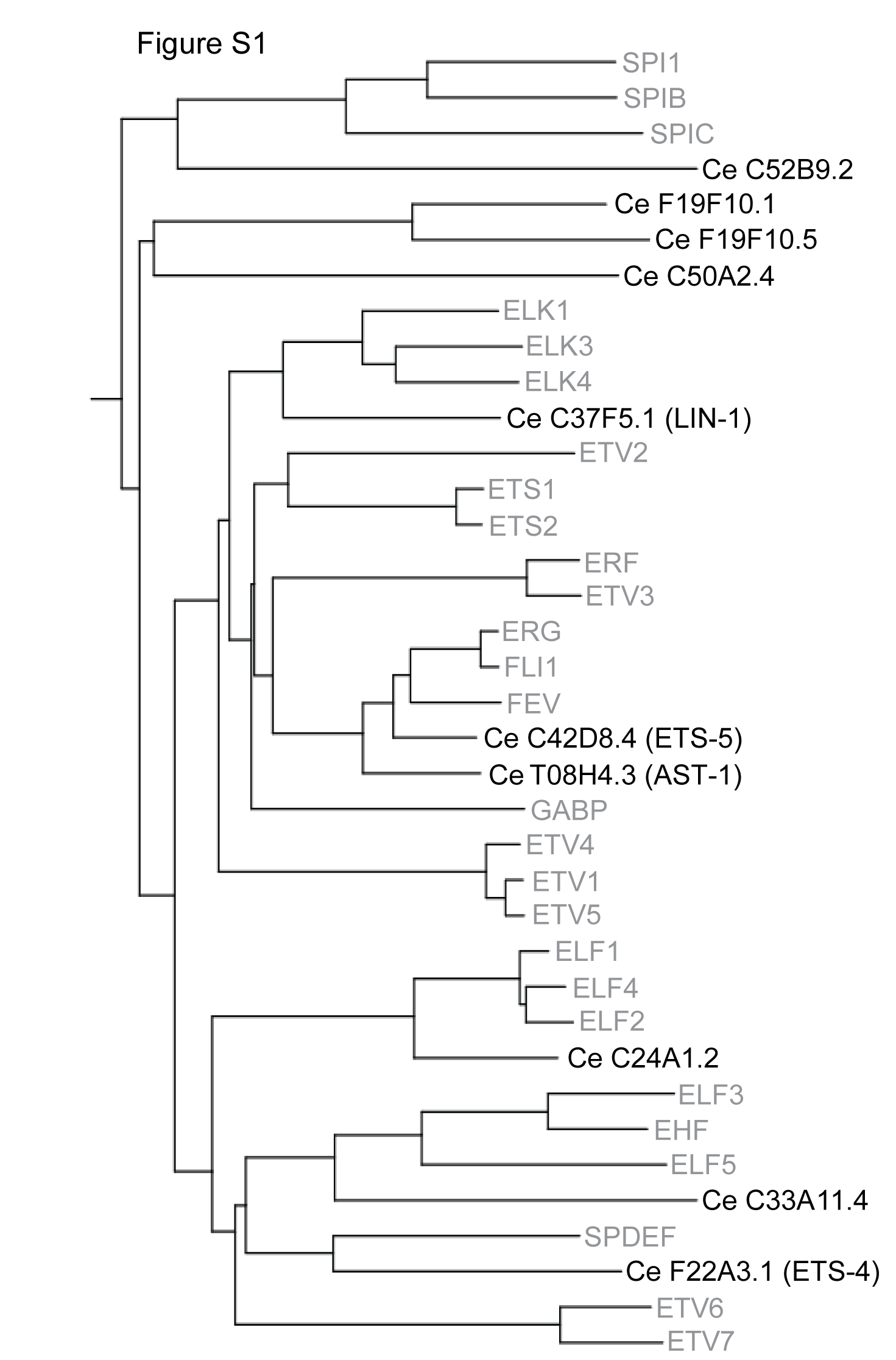

Supplement: Figure S1 — Phylogenetic analysis of ETS domain sequences. The protein sequences of 27 H. sapiens (gray) and 10 C. elegans ETS domains (Ce) were aligned using ClustalW (version 1.83). DRAWGRAM (from phylip version 3.66) was used to construct a dendrogram of the aligned sequences. The horizontal length of the branches predicts the evolutionary distance between the genes. (9.87 MB TIF) [file pgen.1001125.s001.tif]

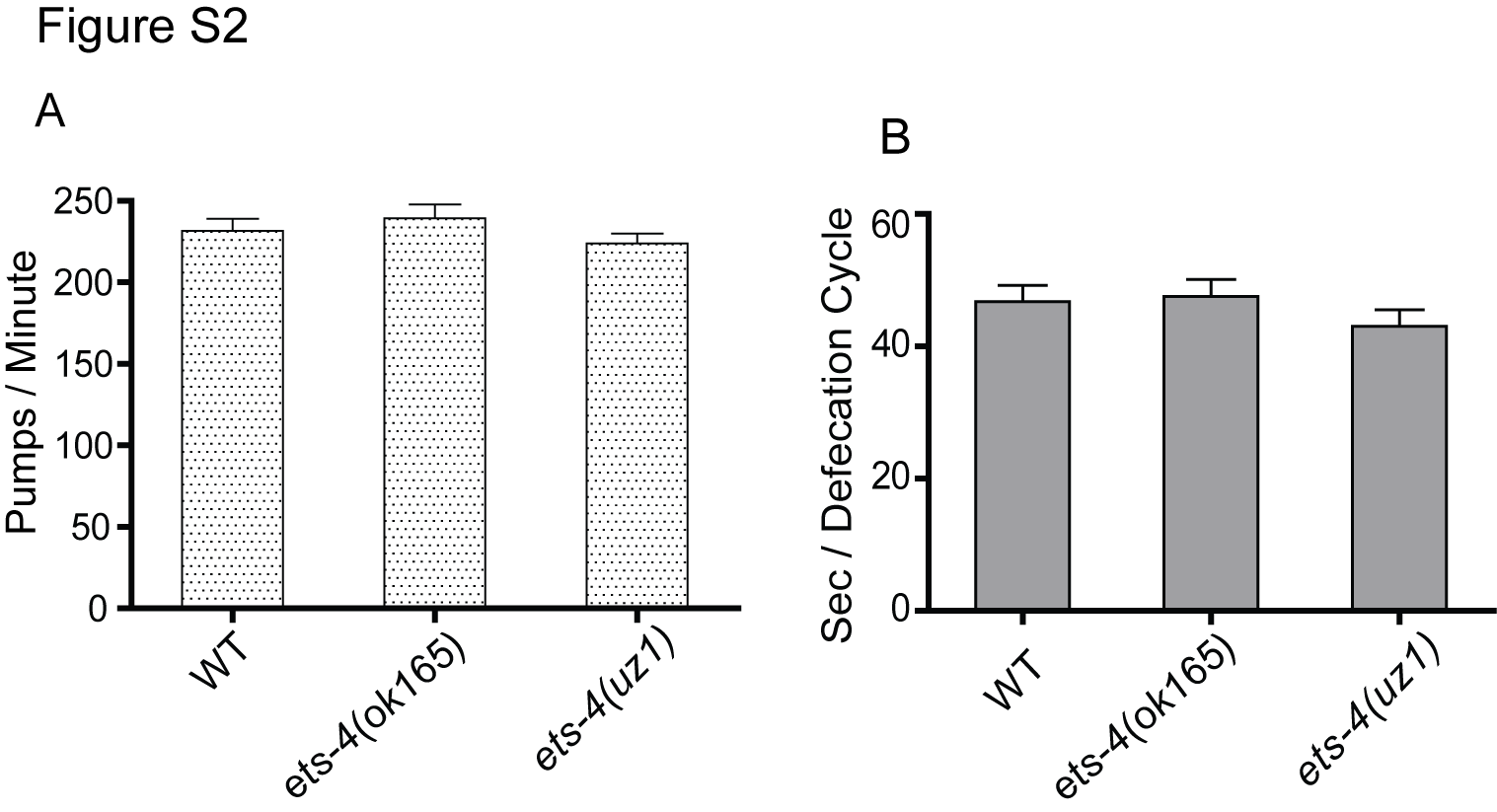

Supplement: Figure S2 — ets-4 mutations do not alter feeding rate or defecation rate. (A) Pharyngeal pumping rates (pumps/minute) were counted for wild-type (WT) (n = 11), ets-4(ok165) (n = 10) and ets-4(uz1) (n = 12) worms (mean ± SEM). (B) Mean defecation cycle periods (seconds/cycle) of wild-type (WT), ets-4(ok165) and ets-4(uz1) worms (mean ± SEM). At least 10 worms per genotype were observed for 10 cycles each. (3.76 MB TIF) [file pgen.1001125.s002.tif]

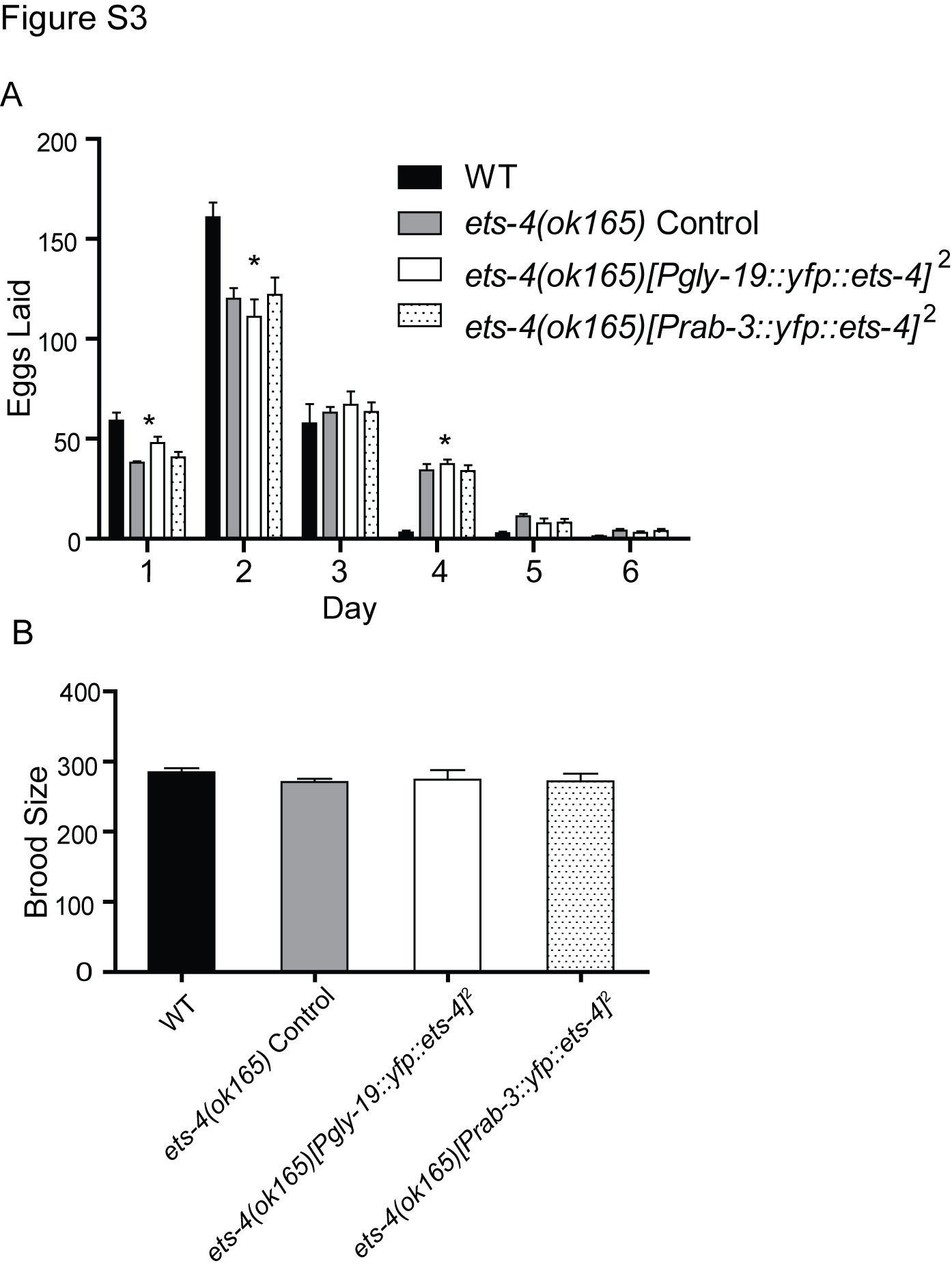

Supplement: Figure S3 — Tissue-specific expression of ETS-4 does not affect the egg-laying rate or brood size of ets-4(ok165) animals. (A) The number of eggs laid (mean ± SEM) during each day of the egg-laying period. The egg-laying rate of wild-type (WT) worms, ets-4(ok165) Control and that of strains with tissue-specific expression of ets-4 in intestinal cells, ets-4(ok165);[Pgly-19::yfp::ets-4], or neurons, ets-4(ok165);[Prab-3::yfp::ets-4] at 20°C. * indicates p<0.001, comparing ets-4(ok165) transgenic lines to WT. (B) Total number of progeny (brood size) was counted for the genotypes indicated at 20°C. The average brood size (mean ± SEM) of ets-4(ok165) Control, ets-4(ok165);[Pgly-19::yfp::ets-4] and ets-4(ok165);[Prab-3::yfp::ets-4] are indicated. (7.80 MB TIF) [file pgen.1001125.s003.tif]

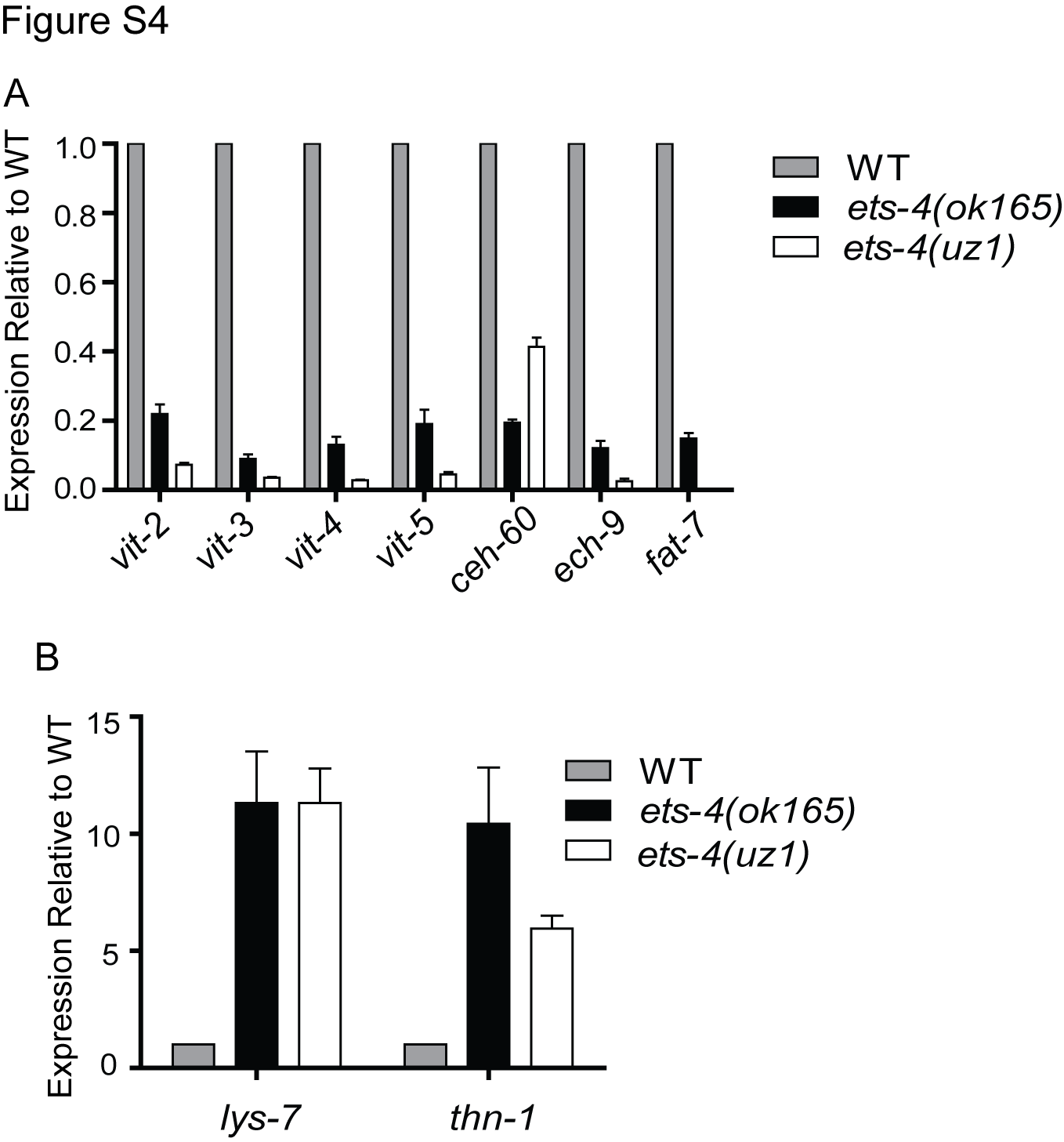

Supplement: Figure S4 — Expression levels of selected genes in ets-4(ok165), ets-4(uz1) and wild-type worms. Gene expression quantified by real-time PCR in ets-4(ok165) and ets-4(uz1) mutant worms relative to wild-type (WT). Error bars represent standard error. (A) Expression of genes significantly down-regulated in ets-4(ok165) worms (shown are vit-2, vit-3, vit-4, vit-5, ceh-60, ech-9 and fat-7) and ets-4(uz1) worms (shown are vit-2, vit-3, vit-4, vit-5, ceh-60 and ech-9) relative to WT. (B) Expression of genes significantly up-regulated in ets-4(ok165) and ets-4(uz1) worms (shown are lys-7 and thn-1) relative to WT. (5.96 MB TIF) [file pgen.1001125.s004.tif]

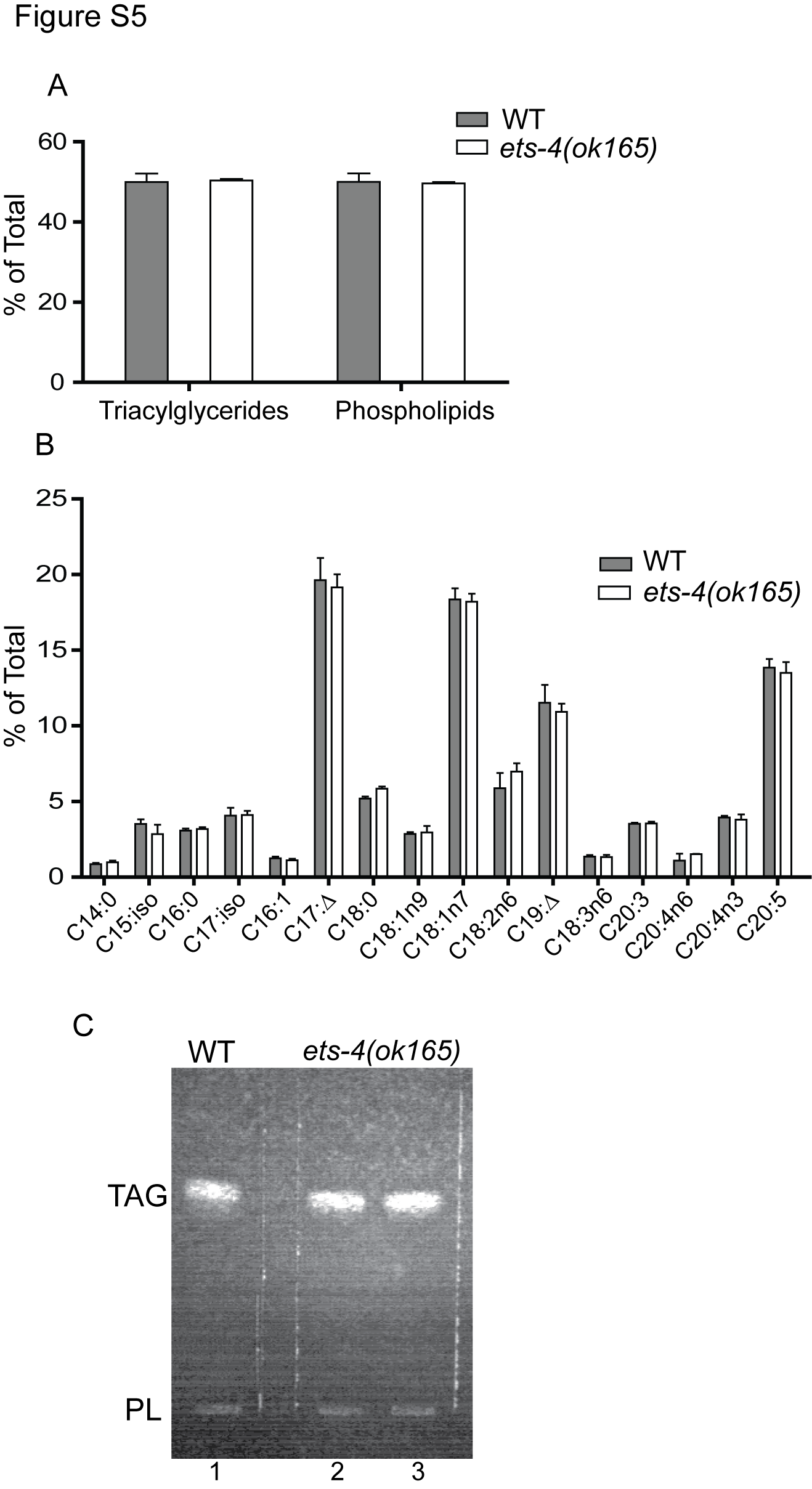

Supplement: Figure S5 — Lipid levels and fatty acid composition is unaltered in ets-4(ok165) worms. (A) Relative abundance of triacylglycerides and phospholipids expressed as percentage of total fatty acids (±standard error) as determined by gas chromatography. (B) Relative abundance of selected fatty acid species expressed as percentage of total fatty acids (± standard error) of WT and ets-4(ok165) worms as determined by gas chromatography. (C) TLC analysis of lipids extracted from wild-type (WT) (lane 1) and ets-4(ok165) animals (replicates in lanes 2 and 3). TAG, triacylglycerides; PL, phospholipids. (9.58 MB TIF) [file pgen.1001125.s005.tif]

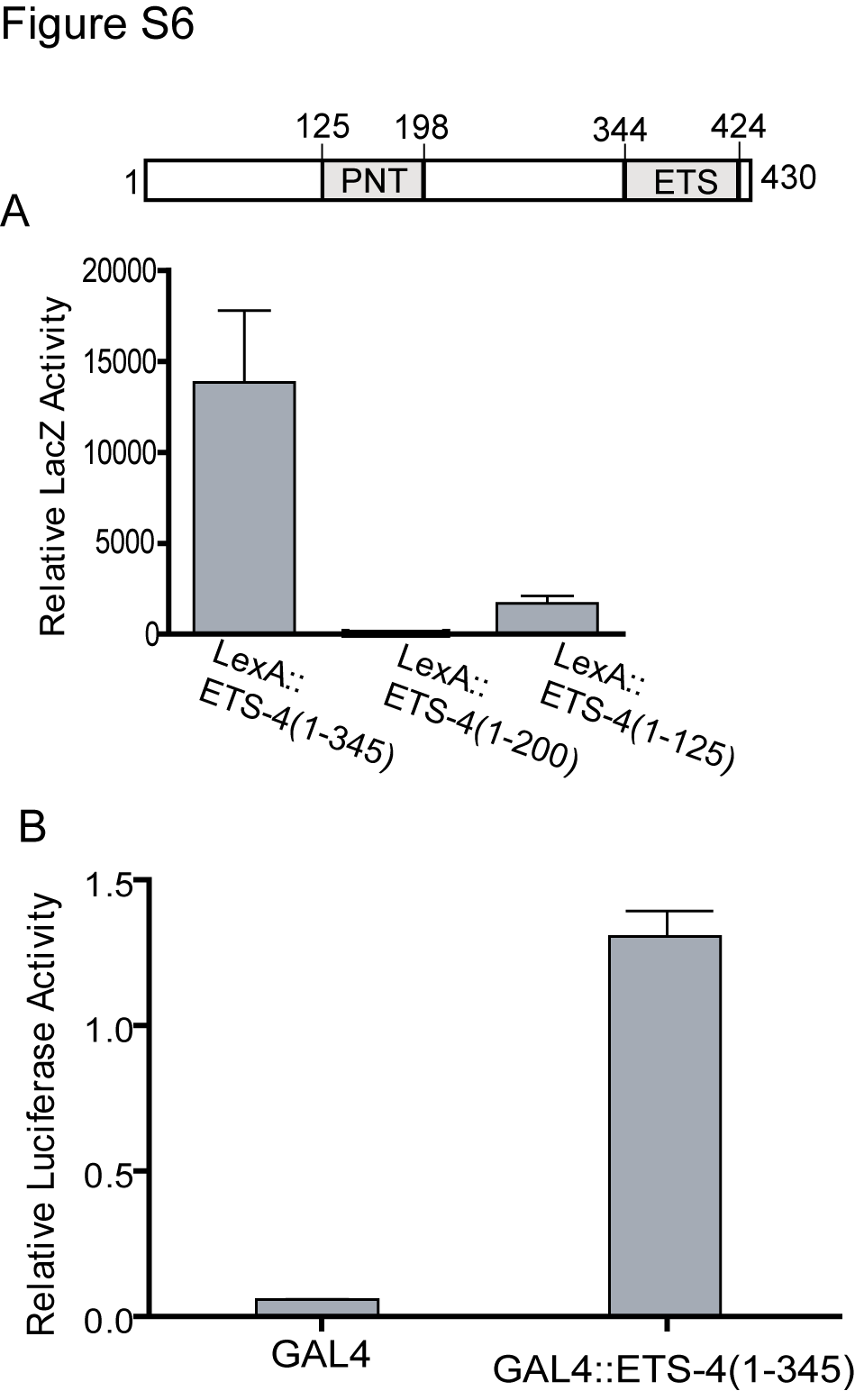

Supplement: Figure S6 — Transcriptional activity of ETS-4. (A) Transcriptional activity of ETS-4 in S. cerevisiae. An yeast strain that contains an integrated LacZ reporter with 8 LexA binding sites was transformed with the indicated LexA::ETS-4 fusions. Cells lysates were subjected to a colorimetric assay to assess β-galactosidase activity (LacZ activity). The relative LacZ activity was calculated by normalizing the LacZ values to that of control strains transformed with the LexA DNA-binding domain. The expression levels of the LexA proteins were comparable (data not shown). Inset is a schematic of ETS-4. (B) Transient expression assays performed in NIH3T3 cells. Luciferase activity was measured from cells transfected with a GAL4-dependent luciferase reporter and expression vectors for GAL4 DNA-binding domain alone or as a fusion to ETS-4. Relative luciferase activity (RLA) was calculated as the ratio of firefly luciferase activity to Renilla luciferase activity (mean± SEM). The expression levels of the GAL4DBD proteins were comparable (data not shown). (4.44 MB TIF) [file pgen.1001125.s006.tif]

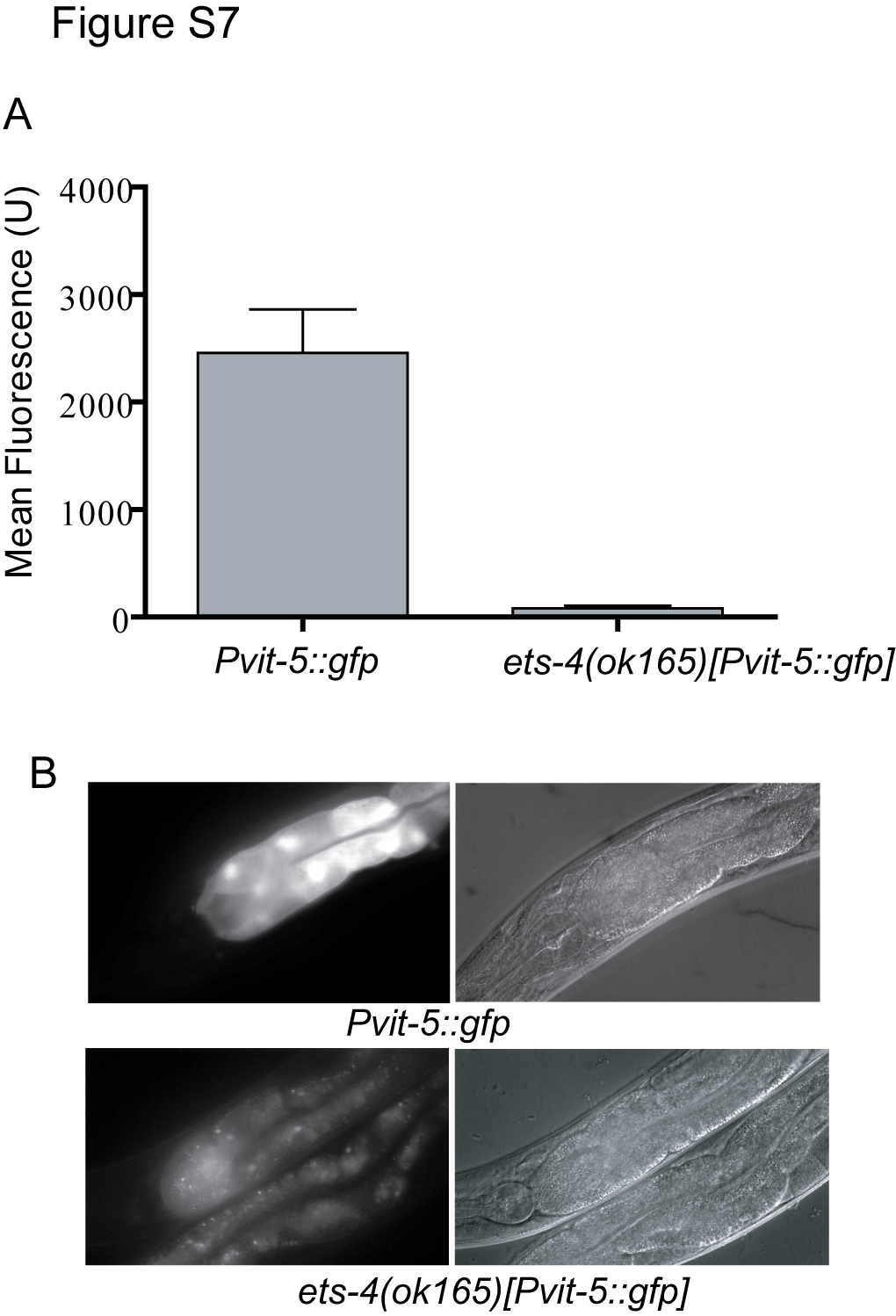

Supplement: Figure S7 — Reduced expression of Pvit-5::gfp in ets-4(ok165) worms. (A) Quantification of Pvit-5::gfp expression in synchronized 1-day old wild-type (WT) and ets-4(ok165) adult hermaphrodites using ImageJ (version 1.36b) software. The mean fluorescence (±SEM) of ets-4(ok165)[Pvit-5::gfp] worms was measured in a fixed area at the start of the intestine and compared to that of Pvit-5::gfp worms in an identical area. (B) Representative example of the reduced Pvit-5::gfp expression in ets-4(ok165) worms compared to wild-type (WT) worms. (4.60 MB TIF) [file pgen.1001125.s007.tif]

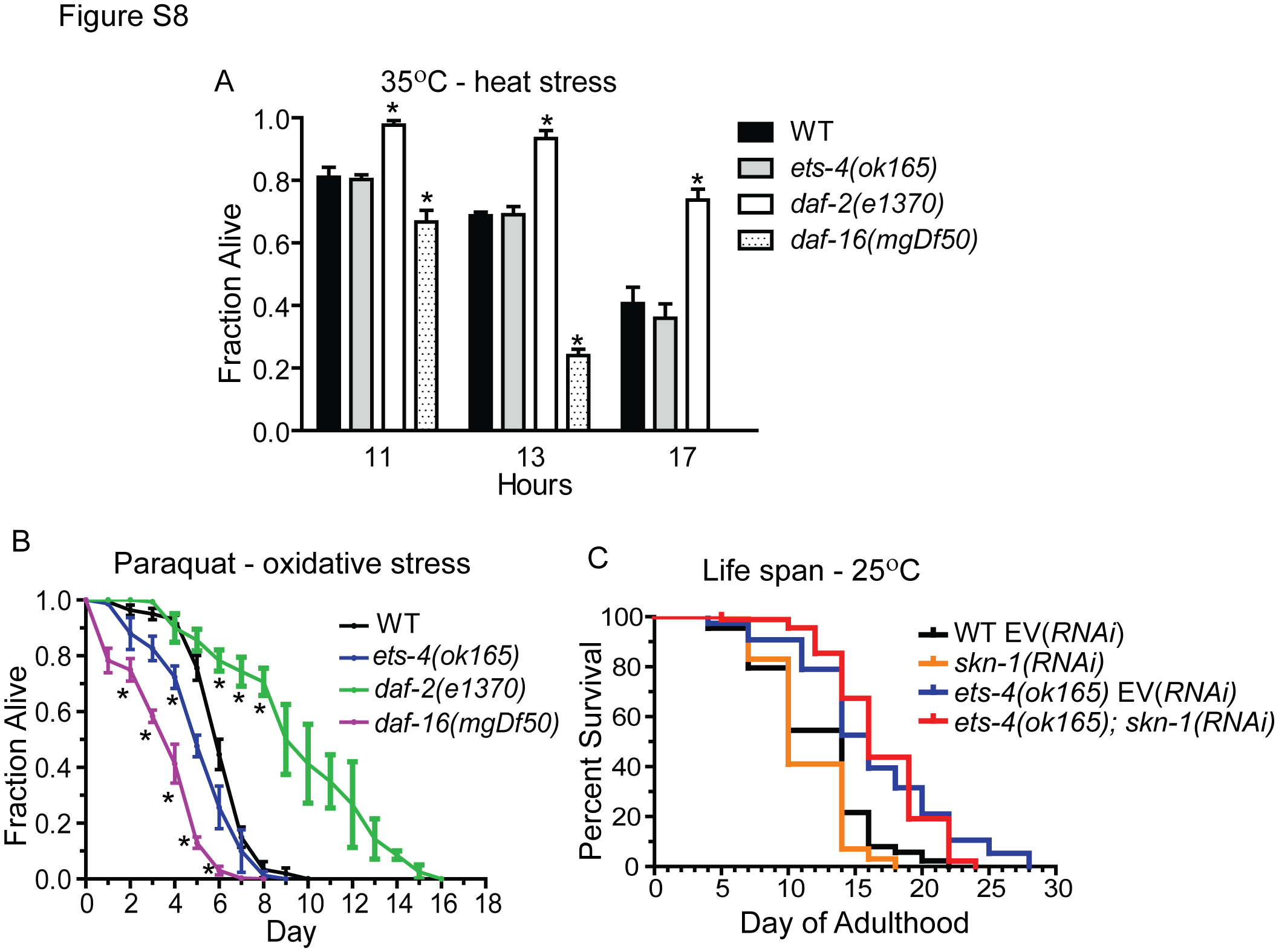

Supplement: Figure S8 — Stress resistance of ets-4(ok165) animals and genetic interaction between ets-4 and skn-1 for life span. (A) Survival kinetics of animals under heat stress conditions (35°C). Fraction of animals alive at different time points (hours) after a shift to 35°C (mean ± SEM) is plotted. * indicates p<0.05, compared to WT. (B) Survival kinetics of animals in the presence of paraquat (oxidative stress conditions - See Text S1). Fraction of animals alive (mean ± SEM) is plotted. * indicates p<0.05, compared to WT. (C) Survival curves for wild-type (WT) and ets-4(ok165) worms grown at 25°C and subjected to skn-1(RNAi) or an empty vector (EV) control RNAi starting at the L4 stage. See Table S1 for mean life span, statistical analyses and data from additional trials. (10.00 MB TIF) [file pgen.1001125.s008.tif]
