# Supplementary material for: ETS-4 Is a Transcriptional Regulator of Life Span in Caenorhabditis elegans
Source: PLoS Genet. 2010 Sep 16;6(9):e1001125. doi: 10.1371/journal.pgen.1001125 (PMC2940738; doi:10.1371/journal.pgen.1001125)
Supplement: Table S1 — Summary of Data from Repeat Trials of Life Span Analysis for ets-4(ok165) and ets-4(uz1) Mutant Worms. (0.11 MB DOC) [file pgen.1001125.s009.doc]

Table S1. Summary of Data from Repeat Trials of Life Span Analysis for *ets-4(ok165)* and *ets-4(uz1)* Mutant Worms^a^.

| **Genotype**^b^ | **Mean Life Span±SEM**^b^  **(Days)** | **N**^b,c^ | **p-value**^b^  **Vs WT controls** | **Vs Strain**^b^ | **p-value**^b^ |
| --- | --- | --- | --- | --- | --- |
| WT ^d^ | 11.4 ± 0.3 | 102 |  |  |  |
| *ets-4(ok165)* | 18.6 ± 0.5 | 90 | < 0.0001 |  |  |
| *ets-4(uz1)* | 16.6 ± 0.5 | 82 | < 0.0001 |  |  |
| WT 20°C | 15.3 ± 0.6 | 96 |  |  |  |
| *ets-4(ok165)*  20°C | 28.6 ± 0.9 | 66 | < 0.0001 |  |  |
| *ets-4(uz1)* 20°C | 22.3 ± 0.8 | 73 | < 0.0001 |  |  |
| WT EV(*RNAi*)^e^ | 12.7 ± 0.5 | 86 |  |  |  |
| *ets-4(RNAi)* | 14.2 ± 0.5 | 94 | 0.0092 |  |  |
| WT EV(*RNAi*) | 12.5 ± 0.4 | 89 |  |  |  |
| *ets-4(RNAi)* | 16.7 ± 0.5 | 91 | < 0.0001 |  |  |
| WT EV*(RNAi)* | 13.4 ± 0.6 | 53 |  |  |  |
| *ets-4(ok165)* EV*(RNAi)* | 18.0 ± 0.3 | 86 | < 0.0001 |  |  |
| *daf-16(RNAi)* | 11.3 ± 0.4 | 75 | < 0.0001 |  |  |
| *daf-16(RNAi); ets-4(ok165)* | 11.1 ± 0.3 | 83 | < 0.0001 | *ets-4(ok165)*EV*(RNAi)* | < 0.0001 |
|  |  |  |  | *daf-16(RNAi)* | 0.2(ns)^f^ |
| WT EV*(RNAi)* | 12.4 ± 0.4 | 83 |  |  |  |
| *ets-4(ok165)* EV*(RNAi)* | 15.2 ± 0.2 | 98 | < 0.0001 |  |  |
| *daf-16(RNAi)* | 10.4 ± 0.1 | 62 | < 0.0001 |  |  |
| *daf-16(RNAi); ets-4(ok165)* | 10.8 ± 0.7 | 73 | < 0.0001 | *ets-4(ok165)*EV*(RNAi)* | < 0.0001 |
|  |  |  | < 0.0001 | *daf-16(RNAi)* | 0.2(ns) |
| WT EV*(RNAi)* | 13.5 ± 0.5 | 82 |  |  |  |
| *ets-4(ok165)* EV*(RNAi)* | 18.1 ± 0.5 | 83 | < 0.0001 |  |  |
| *daf-2(RNAi)* | 20.6 ± 1.1 | 79 | < 0.0001 |  |  |
| *daf-2(RNAi); ets-4(ok165)* | 26.1 ± 0.7 | 82 | < 0.0001 | *ets-4(ok165)* EV*(RNAi)* | < 0.0001 |
|  |  |  |  | *daf-2(RNAi)* | < 0.0001 |
| *akt-1/2(RNAi)* | 15.0 ± 0.6 | 75 | 0.0127 |  |  |
| *akt-1/2(RNAi)*; *ets-4(ok165)* | 21.0 ± 0.5 | 96 | < 0.0001 | *ets-4(ok165)* EV*(RNAi)* | < 0.0001 |
|  |  |  |  | *akt-1/2(RNAi)* | < 0.0001 |
| WT EV*(RNAi)* | 12.7 ± 0.3 | 61 |  |  |  |
| *ets-4(ok165)* EV*(RNAi)* | 17.9 ± 0.4 | 102 | < 0.0001 |  |  |
| *daf-2(RNAi)* | 22.0 ± 1.0 | 50 | < 0.0001 |  |  |
| *daf-2(RNAi); ets-4(ok165)* | 27.0 ± 0.6 | 105 | < 0.0001 | *ets-4(ok165)* EV*(RNAi)* | < 0.0001 |
|  |  |  |  | *daf-2(RNAi)* | < 0.0001 |
| *akt-1/2(RNAi)* | 19.2 ± 0.3 | 122 | < 0.0001 |  |  |
| *akt-1/2(RNAi)*; *ets-4(ok165)* | 24.0 ± 0.5 | 90 | < 0.0001 | *ets-4(ok165)* EV*(RNAi)* | < 0.0001 |
|  |  |  |  | *akt-1/2(RNAi)* | < 0.0001 |
|  |  |  |  |  |  |
| **Genotype**^b^ | **Mean Life Span±SEM**^b^  **(Days)** | **N**^b,c^ | **p-value**^b^  **Vs WT controls** | **Vs Strain**^b^ | **p-value**^b^ |
| *ets-4(ok165)* Control^g^ | 17.4 ± 0.5 | 61 |  |  |  |
| *ets-4(ok165)[Pgly-19::yfp::ets-4]*^1^ | 14.3 ± 0.4 | 76 |  | *ets-4(ok165)* Control | < 0.0001 |
| *ets-4(ok165)[Pgly-19::yfp::ets-4]*^2^ | 14.8 ± 0.8 | 31 |  | *ets-4(ok165)* Control | 0.005 |
| *ets-4(ok165)[Prab-3::yfp::ets-4]*^1^ | 17.1 ± 0.3 | 73 |  | *ets-4(ok165)* Control | 0.2(ns) |
| *ets-4(ok165)[Prab-3::yfp::ets-4]*^2^ | 17.2 ± 0.3 | 94 |  | *ets-4(ok165)* Control | 0.7(ns) |
| WT EV*(RNAi)* | 12.2 ± 0.4 | 88 |  |  |  |
| *ets-4(ok165)* EV*(RNAi)* | 16.3 ± 0.6 | 76 | < 0.0001 |  |  |
| *skn-1(RNAi)* | 11.3 ± 0.3 | 100 | 0.0171 |  |  |
| *skn-1(RNAi); ets-4(ok165)* | 16.8 ± 0.4 | 89 | < 0.0001 | *ets-4(ok165)*EV*(RNAi)* | 0.8(ns) |
|  |  |  |  | *skn-1(RNAi)* | < 0.0001 |
| WT EV*(RNAi)* | 12.9 ± 0.3 | 103 |  |  |  |
| *ets-4(ok165)* EV*(RNAi)* | 16.7 ± 0.7 | 80 | < 0.0001 |  |  |
| *skn-1(RNAi)* | 11.8 ± 0.5 | 79 | 0.2(ns) |  |  |
| *skn-1(RNAi); ets-4(ok165)* | 17.6 ± 0.5 | 92 | < 0.0001 | *ets-4(ok165)*EV*(RNAi)* | 0.8(ns) |
|  |  |  |  | *skn-1(RNAi)* | < 0.0001 |

^a^Independent repeats of each life span experiment were performed. Data from repeat trials are separated by dashed lines.

^b^Life span data sets within each panel of this table were done in parallel and statistical analyses was done within the data set.

^c^Number of worms scored.

^d^WT refers to wild-type.

^e^EV refers to empty vector.

^f^ns indicates non-significant p-values that are greater than 0.05.

^g^*ets-4(ok165)* Control refers to injection control strain. Strain details in Text S1.

^1,2^Refer to independent lines.
