# Supplementary material for: ETS-4 Is a Transcriptional Regulator of Life Span in Caenorhabditis elegans
Source: PLoS Genet. 2010 Sep 16;6(9):e1001125. doi: 10.1371/journal.pgen.1001125 (PMC2940738; doi:10.1371/journal.pgen.1001125)
Supplement: Table S2 — Genes with Altered Expression in ets-4(ok165) Compared to Wild-type (WT) Worms. (0.09 MB DOC) [file pgen.1001125.s010.doc]

Table S2. Genes with Altered Expression in *ets-4(ok165)* Compared to Wild-type (WT) Worms.

| **Genes Downregulated in *ets-4(ok165)*** | | **Genes Upregulated in *ets-4(ok165)*** | |
| --- | --- | --- | --- |
| **Gene** | ***ets-4(ok165)* / WT Ratio** | **Gene** | ***ets-4(ok165)* / WT Ratio** |
| F21F8.4.1 | 0.0545 | F45D11.14 | 5.6388 |
| F56A4.3 | 0.0581 | C02A12.4.1 | 5.522 |
| F15E11.10 | 0.0648 | H16D19.1 | 5.3963 |
| F56A4.9 | 0.0828 | T14E8.2 | 5.1154 |
| F01G10.3 | 0.0938 | D1014.7 | 4.8928 |
| C49D10.4 | 0.0971 | Y38E10A.19 | 4.0435 |
| F59A1.9 | 0.1139 | F45D11.1.1 | 3.9279 |
| F22A3.1 | 0.1217 | F28D1.4 | 3.8969 |
| Y46C8AL.3 | 0.1404 | T10D4.4 | 3.8821 |
| Y48G8AL.11 | 0.2007 | D1014.6 | 3.8787 |
| D2063.2 | 0.2039 | T23F1.5 | 3.7753 |
| F59D8.1.1 | 0.2234 | Y25C1A.11 | 3.7685 |
| C01B4.5 | 0.2335 | C49F5.1.2 | 3.727 |
| F59D8.2 | 0.2374 | F17E9.11 | 3.6771 |
| F56A4.2 | 0.2453 | F45D11.4 | 3.6578 |
| B0250.7 | 0.2507 | C01G10.15 | 3.6458 |
| C04F6.1 | 0.2568 | F55G11.4 | 3.6239 |
| Y45G12C.3 | 0.2775 | F10G2.3 | 3.4655 |
| F57G4.4 | 0.2791 | T22H6.5 | 3.4371 |
| Y48G9A.10 | 0.29 | C14C6.2 | 3.4294 |
| F56H6.5 | 0.2996 | F28D1.3 | 3.3188 |
| C16C4.4 | 0.3012 | K02E7.6 | 3.3039 |
| R09B5.9 | 0.303 | E03H4.10 | 3.2626 |
| F58E6.7 | 0.3085 | Y38E10A.18 | 3.2395 |
| C01B4.9 | 0.3096 | F47H4.2 | 3.1877 |
| F22A3.4 | 0.313 | F26D2.16 | 3.1795 |
| C16C4.15 | 0.3155 | ZK666.7 | 3.1757 |
| F44C8.7 | 0.3203 | F28D1.5 | 3.1686 |
| F56A4.10 | 0.3208 | Y59E9AR.6 | 3.1618 |
| ZK488.4 | 0.3241 | F35D11.9 | 3.0998 |
| Y102A5C.15 | 0.3281 | C50F7.9 | 3.0585 |
| R09B5.3.2 | 0.3321 | Y74C10AR.2 | 3.0469 |
| F10D2.9 | 0.3378 | B0365.6 | 3.0377 |
| F21E9.3 | 0.3404 | Y73F4A.3.1 | 2.956 |
| B0218.8 | 0.3419 | F15E11.15.1 | 2.8487 |
| ZK816.5 | 0.3435 | F21C10.8a | 2.8185 |
| C50E3.12 | 0.3448 | T16G1.4 | 2.7025 |
| Y46H3A.2 | 0.3464 | R13H4.3 | 2.6947 |
| T27E4.3 | 0.3528 | C01G10.4 | 2.6855 |
| T09F5.9 | 0.355 | Y38E10A.25 | 2.6671 |
| Y38H6C.21 | 0.3564 | C09B8.3 | 2.661 |
| C30G12.2 | 0.3567 | R11G11.7 | 2.6542 |
| F42G8.7 | 0.3627 | T15D6.11 | 2.6509 |
| F02E8.4 | 0.3639 | C32H11.13 | 2.598 |
| F57B9.3 | 0.3641 | Y71G12B.18 | 2.5873 |
| C09B8.4 | 0.3647 | F14F8.8 | 2.585 |
| T06D4.1 | 0.3662 | E03H4.4 | 2.5536 |
| F28B12.2b.1 | 0.3665 | F49E11.10 | 2.5361 |
| T07D10.3 | 0.3666 | T15D6.8 | 2.5224 |
| F15E6.4 | 0.3739 | F15E11.12 | 2.4908 |
| R03D7.2 | 0.3745 | T22B11.4b | 2.4786 |
| ZC53.7 | 0.3801 | K02E2.7 | 2.4331 |
| C42D4.2 | 0.3823 | K02E2.4 | 2.4329 |
| C16C4.5 | 0.3841 | F41D3.10 | 2.4324 |
| C56E10.1 | 0.3881 | ZK218.5 | 2.3983 |
| C15H11.3 | 0.3903 | T23F4.3 | 2.3532 |
| C23H5.3 | 0.3918 | F28G4.1 | 2.2296 |
| C13A2.3 | 0.3931 |  |  |
| C01B4.8 | 0.3937 |  |  |
| F58B3.3 | 0.3944 |  |  |
| C54D1.2 | 0.3957 |  |  |
| R07B5.3 | 0.396 |  |  |
| F36H5.10 | 0.3967 |  |  |
| Y19D10A.4 | 0.4002 |  |  |
| C27C7.1 | 0.4031 |  |  |
| F46F5.6 | 0.4047 |  |  |
| T08A9.7.1 | 0.4078 |  |  |
| R119.2 | 0.4095 |  |  |
| C45G7.2 | 0.4118 |  |  |
| T04C9.3 | 0.412 |  |  |
| F58B3.2 | 0.4121 |  |  |
| F46H5.8 | 0.4185 |  |  |
| F58B3.1 | 0.42 |  |  |
| T22B7.7 | 0.4236 |  |  |
| C17C3.12c.1 | 0.424 |  |  |
| W06A11.4 | 0.4243 |  |  |
| R13H4.8 | 0.426 |  |  |
| C43D7.4 | 0.4285 |  |  |
| Y57G11B.5 | 0.432 |  |  |
| Y53F4B.8 | 0.4346 |  |  |
| K04A8.5 | 0.4349 |  |  |
| R08F11.1 | 0.4373 |  |  |
| C42D8.2.1 | 0.4379 |  |  |
| C48B4.1.2 | 0.4382 |  |  |
| C32B5.9 | 0.443 |  |  |
| Y45G12C.7 | 0.4471 |  |  |
| F22A3.6a | 0.4502 |  |  |
| C54C8.2 | 0.4513 |  |  |
| F15B9.1 | 0.4541 |  |  |
