# Supplementary material for: ETS-4 Is a Transcriptional Regulator of Life Span in Caenorhabditis elegans
Source: PLoS Genet. 2010 Sep 16;6(9):e1001125. doi: 10.1371/journal.pgen.1001125 (PMC2940738; doi:10.1371/journal.pgen.1001125)
Supplement: Table S3 — Genes with Altered Expression in ets-4(uz1) compared to Wild-type (WT) Worms. (0.26 MB DOC) [file pgen.1001125.s011.doc]

Table S3. Genes with Altered Expression in *ets-4(uz1)* compared to Wild-type (WT) Worms.

| **Genes Downregulated in *ets-4(uz1)*** | | **Genes Upregulated in *ets-4(uz1)*** | |
| --- | --- | --- | --- |
| **Gene** | ***ets-4(uz1)*/WT Ratio** | **Gene** | ***ets-4(uz1)*/WT Ratio** |
| F36H12.13 | 0.0448 | F15B9.1 | 16.0875 |
| F36H12.14 | 0.0526 | F17E9.11 | 14.6689 |
| F21F8.4.1 | 0.0559 | F48C1.8 | 14.1118 |
| F01G10.3 | 0.0657 | F48C1.9 | 12.9833 |
| Y57G11B.5 | 0.0877 | F07G11.9 | 10.4713 |
| F55B11.3 | 0.0908 | E03H4.10 | 9.7154 |
| Y62H9A.3 | 0.1005 | F53H2.2 | 9.0974 |
| Y46C8AL.3 | 0.1013 | Y41G9A.5 | 8.9812 |
| ZK1193.1a | 0.1053 | T20D4.12 | 8.7065 |
| Y37D8A.19.2 | 0.1129 | C02A12.4.1 | 8.1054 |
| T15D6.8 | 0.1134 | R09B5.3.2 | 7.6634 |
| F54B11.1 | 0.1136 | Y69H2.3d.2 | 7.6501 |
| F59D8.2 | 0.114 | C45B2.2 | 7.335 |
| T15D6.11 | 0.1151 | Y39H10A.1 | 7.2709 |
| R13H4.8 | 0.1192 | F22B7.4 | 7.0869 |
| F59D8.1.1 | 0.1201 | T28A11.3 | 6.9675 |
| Y62H9A.5 | 0.1202 | B0213.2 | 6.9093 |
| F22A3.1 | 0.1221 | C17B7.9 | 6.4262 |
| D1014.6 | 0.1257 | W09G12.7 | 6.3907 |
| Y62H9A.4 | 0.1301 | F55A12.6 | 6.2824 |
| C04F6.1 | 0.1353 | C37A5.8 | 6.2256 |
| C17C3.12c.1 | 0.1441 | C24G7.1 | 6.1625 |
| C17C3.12a | 0.1491 | ZK899.4 | 6.1019 |
| D1054.11 | 0.1535 | C45B2.3 | 6.1004 |
| F11H8.3 | 0.1587 | B0213.6 | 6.0039 |
| C34F6.3 | 0.1664 | C36C5.14 | 5.9844 |
| Y46C8AL.6 | 0.1683 | K02G10.7b | 5.9834 |
| C27A2.5 | 0.1689 | Y55D5A.6 | 5.7074 |
| F56D6.1 | 0.1689 | T22H6.5 | 5.5859 |
| C16C4.15 | 0.1703 | T28A11.16 | 5.4158 |
| F56D6.2 | 0.171 | Y58A7A.2 | 5.3751 |
| F10F2.9 | 0.1713 | F57G8.7 | 5.2032 |
| K07H8.6b | 0.1717 | C17B7.10 | 5.0877 |
| C16C4.4 | 0.1737 | C14C6.4 | 5.0429 |
| Y113G7A.15 | 0.1757 | Y119D3A.4 | 5.0174 |
| Y62H9A.6 | 0.181 | Y7A5A.3 | 4.9836 |
| C54C8.2 | 0.183 | C01G10.4 | 4.9575 |
| Y45F10C.4 | 0.1848 | F15B9.2 | 4.9174 |
| F57C2.4 | 0.1858 | C17B7.12 | 4.9159 |
| W02D9.7 | 0.1868 | R08E5.4 | 4.915 |
| T24A6.3 | 0.1872 | F35B12.3 | 4.8927 |
| F38A3.1 | 0.1907 | R90.4 | 4.8706 |
| C16C4.5 | 0.1909 | T26E4.5 | 4.8578 |
| K07H8.6c | 0.1915 | B0213.3 | 4.8264 |
| E02C12.8b | 0.1976 | F14F7.1.1 | 4.8093 |
| ZK617.2 | 0.1976 | C36C5.15 | 4.6736 |
| D2063.2 | 0.2009 | F53B6.9 | 4.5639 |
| K07A1.6 | 0.2015 | Y80D3A.7 | 4.5279 |
| Y22D7AR.10 | 0.2076 | T28A11.20 | 4.523 |
| M18.1 | 0.2119 | ZK666.7 | 4.4705 |
| F15E6.4 | 0.213 | R10H1.4 | 4.4647 |
| F17E9.4 | 0.2138 | C06E4.8 | 4.4174 |
| C25A8.4 | 0.2148 | C45G9.6b | 4.4082 |
| Y69A2AR.25 | 0.2156 | ZK105.5 | 4.2945 |
| D1014.7 | 0.2161 | C54F6.10 | 4.2819 |
| Y51H4A.9 | 0.2185 | R90.3 | 4.2612 |
| Y69A2AR.13 | 0.2191 | C17B7.3 | 4.2108 |
| H13N06.6 | 0.2209 | C36C5.12 | 4.208 |
| T05A10.5 | 0.2222 | T23F1.5 | 4.1821 |
| R03G8.6 | 0.2277 | B0284.2 | 4.1698 |
| Y48A6B.2 | 0.2301 | F10G2.3 | 4.1152 |
| F55B11.2 | 0.2303 | Y53H1B.2 | 4.0974 |
| D1054.10 | 0.237 | F46F2.5 | 4.0952 |
| ZC373.2 | 0.2383 | C34F11.8 | 4.0868 |
| F57A8.8 | 0.2387 | C34G6.2 | 4.0754 |
| C04H5.7 | 0.2425 | F28D1.4 | 4.0538 |
| C30G12.2 | 0.2436 | ZK105.1 | 4.0328 |
| F16G10.9 | 0.2479 | F25H8.5d | 3.9781 |
| ZK287.9 | 0.2507 | W01C9.1 | 3.953 |
| Y45F10C.2 | 0.2511 | F14F8.8 | 3.9513 |
| F43C11.3 | 0.2576 | F53G2.4b.1 | 3.9493 |
| C09B8.4 | 0.2612 | C28C12.4 | 3.9239 |
| C42D8.2.1 | 0.2616 | F28D1.5 | 3.9157 |
| F37B4.7.1 | 0.2616 | W09D12.1.3 | 3.9123 |
| C06A1.6 | 0.2617 | F59C6.6 | 3.9103 |
| T24A6.19 | 0.2633 | F28D1.3 | 3.9041 |
| C26B2.8 | 0.2634 | Y73F4A.2 | 3.9019 |
| F40C5.3 | 0.2648 | B0213.17 | 3.8751 |
| R05A10.7 | 0.267 | Y51A2D.11 | 3.8356 |
| T13F3.6 | 0.2675 | F12A10.7 | 3.8208 |
| Y77E11A.15.1 | 0.2683 | ZK783.1 | 3.8208 |
| F15A2.1 | 0.2691 | R13D11.10 | 3.8108 |
| C44B7.5 | 0.2694 | ZC21.3 | 3.8083 |
| A_12_P106748 | 0.2695 | C54D1.1 | 3.783 |
| F21E9.3 | 0.2696 | T28A11.19 | 3.7764 |
| F22A3.4 | 0.2699 | Y49G5A.1 | 3.7228 |
| F47B7.4 | 0.2715 | C55A1.6 | 3.7226 |
| ZC443.5.2 | 0.2733 | R11G11.7 | 3.7213 |
| Y71H2AM.16 | 0.2747 | C01B4.8 | 3.6878 |
| K10H10.4 | 0.2763 | F01E11.1 | 3.6797 |
| C42D4.2 | 0.2766 | R05H10.6 | 3.6734 |
| C52B9.9 | 0.2789 | C09F12.2 | 3.65 |
| T08A9.7.1 | 0.2803 | C04E12.2 | 3.6108 |
| F54F7.3 | 0.282 | T14B4.6 | 3.5769 |
| F42A10.7.1 | 0.2821 | T26E4.4 | 3.5696 |
| T13B5.4 | 0.2824 | T20D4.11 | 3.5499 |
| C23H5.3 | 0.2827 | C28G1.2 | 3.5498 |
| B0218.8 | 0.2855 | Y73F4A.3.1 | 3.5254 |
| F28F8.2.4 | 0.2858 | ZK218.5 | 3.4996 |
| F23F12.12 | 0.2864 | ZK1290.8 | 3.4986 |
| C08F11.11 | 0.2893 | T23F1.6 | 3.4754 |
| M02D8.4a.1 | 0.2897 | C34C6.7 | 3.453 |
| F42G8.7 | 0.2904 | F08F1.4a | 3.4517 |
| H25K10.1 | 0.2904 | E04F6.8 | 3.4492 |
| T23F6.1 | 0.2929 | C38C3.9 | 3.4278 |
| K11G9.3 | 0.2935 | R13A5.6 | 3.4278 |
| Y46C8AL.5 | 0.2935 | W04G3.8.2 | 3.4158 |
| W06G6.11 | 0.2945 | Y59E9AR.6 | 3.4151 |
| Y60A3A.5 | 0.2974 | ZK1320.4 | 3.415 |
| W05G11.3.1 | 0.2979 | K11G9.5 | 3.401 |
| C10G8.4 | 0.2983 | F26D11.5 | 3.3968 |
| T19H5.1 | 0.2983 | F35F10.4 | 3.3916 |
| C49D10.4 | 0.2987 | H16D19.1 | 3.3822 |
| B0334.13 | 0.2994 | T28A11.18 | 3.3728 |
| C48B4.1.2 | 0.3043 | T23F4.3 | 3.3697 |
| C49G7.5 | 0.3059 | F21H7.12 | 3.3682 |
| T11F9.3 | 0.3065 | Y45G12C.4 | 3.364 |
| C55B7.4b.4 | 0.3066 | T10B10.1 | 3.3626 |
| F58G4.6 | 0.3069 | C01G10.15 | 3.3614 |
| Y39G10AR.6b | 0.3083 | C01G10.6 | 3.3473 |
| W02D3.7 | 0.3092 | F12A10.1 | 3.3378 |
| W02D9.6 | 0.3108 | B0365.6 | 3.3242 |
| F07G6.7 | 0.3109 | F14H12.3 | 3.317 |
| ZK512.7 | 0.3132 | C18C4.1 | 3.3032 |
| W03G11.2 | 0.3133 | C25E10.4 | 3.2905 |
| C10H11.6 | 0.3137 | F44B9.1c | 3.289 |
| E03H4.4 | 0.3139 | C32H11.9 | 3.2851 |
| F49E12.2 | 0.3143 | H12I19.4 | 3.284 |
| Y46C8AL.4 | 0.3182 | W04G3.2.2 | 3.2536 |
| T01B7.8 | 0.3183 | Y38E10A.15 | 3.2509 |
| T07D1.3 | 0.3189 | C54D1.7 | 3.2492 |
| EGAP9.2 | 0.3199 | F55G11.4 | 3.2371 |
| Y46H3A.3 | 0.3207 | C44H4.2 | 3.2358 |
| D1007.13 | 0.3215 | T14B4.7b | 3.2293 |
| K09F5.2 | 0.3216 | F23H12.9 | 3.2224 |
| F47E1.4.1 | 0.3221 | T04F3.3 | 3.2161 |
| F31D5.1 | 0.3231 | F56B3.1 | 3.2151 |
| C35B1.4 | 0.324 | F45E4.5 | 3.1618 |
| B0218.6 | 0.3258 | B0238.12 | 3.1493 |
| T07H3.3 | 0.3266 | F13B9.2 | 3.1233 |
| Y75D11A.5 | 0.3266 | F21C10.8a | 3.1056 |
| F27D9.2.2 | 0.327 | C29E6.1 | 3.0935 |
| R11E3.4 | 0.3271 | F46C8.6 | 3.0854 |
| T12D8.5.1 | 0.3277 | F21H7.3 | 3.07 |
| Y54G2A.8 | 0.328 | F44F4.4.2 | 3.0535 |
| D2096.6 | 0.3295 | C45B11.2 | 3.0495 |
| T08A9.8 | 0.3296 | R11G11.6 | 3.0452 |
| T09F5.9 | 0.3306 | K06G5.2 | 3.043 |
| T01C8.3 | 0.3338 | K09F6.9 | 3.0348 |
| C28G1.1 | 0.3356 | F25D1.3 | 3.0203 |
| C39B5.7 | 0.336 | C15F1.2 | 3.0159 |
| F07H5.8 | 0.3405 | M03B6.3 | 3.0085 |
| ZK488.4 | 0.342 | M7.3.1 | 2.9992 |
| C25D7.4 | 0.3424 | F47E1.2 | 2.989 |
| C32H11.1 | 0.3431 | F12F6.9 | 2.9809 |
| K08D8.5 | 0.3471 | F52B11.3.2 | 2.9758 |
| Y49C4A.8b.2 | 0.349 | F01D4.8 | 2.9681 |
| C40C9.3 | 0.3518 | T21B6.5 | 2.9608 |
| C26G2.2 | 0.353 | K04F1.9 | 2.9301 |
| ZC416.6 | 0.3554 | T14B4.7a.2 | 2.9212 |
| F38A1.14 | 0.3556 | T22H6.3 | 2.9193 |
| Y53F4B.18 | 0.3556 | F11E6.9 | 2.9184 |
| F22D6.10 | 0.3566 | C03G6.5 | 2.9181 |
| T16G1.5 | 0.3569 | T22F7.4 | 2.8929 |
| T06E4.8 | 0.3584 | H41C03.1 | 2.8879 |
| C10H11.4 | 0.3586 | R05H10.1 | 2.8852 |
| F28B12.2b.1 | 0.3594 | W04G3.1 | 2.8845 |
| Y45G12C.1 | 0.3599 | C14C6.2 | 2.8787 |
| T21E8.2 | 0.3606 | W04G3.3 | 2.8765 |
| T28F2.1 | 0.3608 | C05D11.6 | 2.8745 |
| C23H5.8b | 0.3615 | F41E6.6.2 | 2.8744 |
| F57H12.7 | 0.3626 | C01B4.7 | 2.8735 |
| F46G10.6.2 | 0.3632 | T17H7.1.1 | 2.8698 |
| T01G5.2 | 0.3637 | ZK488.10 | 2.8617 |
| R05A10.6 | 0.3641 | F53B2.2 | 2.8552 |
| F48B9.2 | 0.3648 | C02C2.1 | 2.8513 |
| Y87G2A.15 | 0.3651 | K08F4.12 | 2.8391 |
| F43C1.5 | 0.3659 | F36D3.3 | 2.8332 |
| F52B11.4 | 0.3682 | R07B7.11 | 2.8208 |
| F14B8.4 | 0.3693 | F44E2.4.2 | 2.8195 |
| C32D5.6 | 0.3702 | C26F1.2 | 2.8141 |
| F41F3.4 | 0.3712 | VK10D6R.1 | 2.7987 |
| ZK816.5 | 0.3712 | ZC449.2 | 2.7932 |
| T25E4.1 | 0.3722 | C32H11.13 | 2.7921 |
| F45E6.3 | 0.3758 | C38D9.2 | 2.7915 |
| T10E10.4 | 0.376 | M03A1.7 | 2.7901 |
| C54D2.1 | 0.3764 | T10B9.8 | 2.7849 |
| T22B7.3 | 0.377 | R03H4.6 | 2.7774 |
| Y4C6B.6 | 0.377 | C44H4.3 | 2.7724 |
| F18F11.1 | 0.3772 | ZK337.1a | 2.7711 |
| F15E6.8 | 0.3777 | W01F3.2 | 2.767 |
| F57B1.3 | 0.3803 | Y51H7C.2 | 2.7629 |
| D1065.5 | 0.3828 | Y10G11A.3 | 2.7617 |
| F07B10.1 | 0.3835 | C37E2.2 | 2.7604 |
| T10E9.3 | 0.3857 | K01D12.14 | 2.7595 |
| E04F6.15 | 0.3896 | Y41G9A.2 | 2.7569 |
| F47G4.3 | 0.3933 | F47G3.1 | 2.7484 |
| F45E6.4 | 0.3934 | C53B7.3 | 2.7393 |
| F46H5.8 | 0.396 | T01C4.1 | 2.7348 |
| F55G11.5 | 0.3962 | C44C1.6 | 2.7275 |
| F18E3.7a | 0.3966 | F42G9.2 | 2.7247 |
| Y74C9A.1 | 0.3966 | Y9C9A.1 | 2.7219 |
| C04G6.10 | 0.3967 | K07C5.7 | 2.7216 |
| ZK337.5 | 0.3968 | F19H8.2 | 2.7173 |
| ZK892.2 | 0.3981 | W08A12.4 | 2.7149 |
| C07A4.3 | 0.3982 | Y57G11C.20 | 2.7131 |
| C54G6.5.1 | 0.3985 | F01D5.5 | 2.7067 |
| Y51H4A.5 | 0.399 | C54C8.4 | 2.6928 |
| Y70C5A.2.1 | 0.3991 | C14C6.8 | 2.6833 |
| C06B3.3 | 0.4008 | F42A9.6 | 2.6779 |
| F45G2.1 | 0.4023 | ZC449.1 | 2.6777 |
| ZK1240.1 | 0.4026 | Y25C1A.11 | 2.6679 |
| F42G9.9b.1 | 0.403 | Y71F9B.13c.2 | 2.6618 |
| M02G9.3 | 0.4033 | T20D4.15 | 2.6606 |
| C23G10.6 | 0.4055 | Y10G11A.2 | 2.6597 |
| R08B4.5 | 0.4059 | C11H1.1 | 2.6467 |
| F19C7.4 | 0.4061 | T28C6.1 | 2.6459 |
| R03C1.1 | 0.4083 | B0205.4 | 2.6457 |
| F09F7.4a.1 | 0.4092 | F07C4.2 | 2.6387 |
| Y46G5A.19.2 | 0.4092 | Y81G3A.4 | 2.6379 |
| F40F9.9 | 0.4139 | K11G12.1.1 | 2.6349 |
| Y102A11A.7 | 0.4142 | F46F3.3 | 2.6296 |
| T28C12.6 | 0.4143 | F15D3.8 | 2.6253 |
| T26E4.10 | 0.4149 | F46F2.3 | 2.6232 |
| F29D10.2 | 0.4165 | F14F3.4 | 2.6137 |
| C18A11.3 | 0.4182 | F41E7.4 | 2.6095 |
| W06H3.3 | 0.4197 | ZC328.1.1 | 2.6055 |
| R05A10.5 | 0.4218 | C17D12.3 | 2.6047 |
|  |  | K07C6.3 | 2.5964 |
|  |  | Y110A2AL.3 | 2.5909 |
|  |  | T28B4.3.1 | 2.588 |
|  |  | M60.4b.2 | 2.5876 |
|  |  | R90.2 | 2.5826 |
|  |  | F07C4.9 | 2.5819 |
|  |  | F53B3.6 | 2.5807 |
|  |  | C02E7.7 | 2.5636 |
|  |  | F56A4.3 | 2.5421 |
|  |  | F41G4.8 | 2.534 |
|  |  | T19C3.9 | 2.5262 |
|  |  | F47H4.2 | 2.5245 |
|  |  | C06E1.8 | 2.5201 |
|  |  | T26C5.2.2 | 2.5198 |
|  |  | BE0003N10.3 | 2.5196 |
|  |  | W02H5.1 | 2.517 |
|  |  | C44F1.3 | 2.5166 |
|  |  | K02C4.2 | 2.5135 |
|  |  | Y45F10B.1 | 2.5125 |
|  |  | Y37A1B.7 | 2.5081 |
|  |  | C29E4.1 | 2.505 |
|  |  | M02B7.6 | 2.4979 |
|  |  | F48E3.8a | 2.4976 |
|  |  | B0511.1 | 2.4947 |
|  |  | F11A5.8 | 2.491 |
|  |  | Y6D1A.2 | 2.4862 |
|  |  | ZK1251.2 | 2.4748 |
|  |  | B0238.13 | 2.4697 |
|  |  | C50F2.7 | 2.4611 |
|  |  | F15E11.10 | 2.4593 |
|  |  | C45B2.8 | 2.4589 |
|  |  | Y45G12C.12 | 2.4569 |
|  |  | R13H4.3 | 2.4541 |
|  |  | Y37D8A.3 | 2.4534 |
|  |  | T12E12.6 | 2.4424 |
|  |  | C54F6.5 | 2.4396 |
|  |  | Y55F3AM.11 | 2.4383 |
|  |  | F47B10.5 | 2.435 |
|  |  | T21C9.8.1 | 2.4321 |
|  |  | Y43C5A.3 | 2.4319 |
|  |  | C14C6.12 | 2.4294 |
|  |  | C50F2.6b.1 | 2.4267 |
|  |  | Y73E7A.8 | 2.4209 |
|  |  | F14D7.5 | 2.419 |
|  |  | C07B5.2 | 2.4174 |
|  |  | Y51A2D.1 | 2.4154 |
|  |  | C34F6.1 | 2.4132 |
|  |  | C31H2.2 | 2.4123 |
|  |  | F49H6.5 | 2.4122 |
|  |  | T22A3.8 | 2.4114 |
|  |  | F52E4.5 | 2.4099 |
|  |  | K11G9.6 | 2.4099 |
|  |  | T06E6.5 | 2.4063 |
|  |  | H03E18.1 | 2.4044 |
|  |  | F17A9.3 | 2.4024 |
|  |  | R09B5.8 | 2.4012 |
|  |  | C17B7.2 | 2.4007 |
|  |  | ZK1248.16 | 2.3987 |
|  |  | ZK328.8 | 2.3985 |
|  |  | Y39A1A.19 | 2.3952 |
|  |  | F26D11.2 | 2.3931 |
|  |  | Y43F8B.7 | 2.3884 |
|  |  | F23D12.7 | 2.3822 |
|  |  | Y19D10A.4 | 2.3779 |
|  |  | T21D12.2 | 2.3764 |
|  |  | K02E10.4 | 2.3753 |
|  |  | H14E04.1 | 2.3739 |
|  |  | F08F1.5 | 2.3665 |
|  |  | Y65B4BR.2 | 2.3651 |
|  |  | B0564.3.1 | 2.365 |
|  |  | Y57G11C.41 | 2.3604 |
|  |  | F07C4.7 | 2.3593 |
|  |  | ZK1290.12 | 2.3471 |
|  |  | E04F6.9 | 2.344 |
|  |  | K02E7.10 | 2.3419 |
|  |  | C26D10.5b | 2.3367 |
|  |  | F16F9.2 | 2.3356 |
|  |  | F55F8.1.1 | 2.3347 |
|  |  | T22B3.1 | 2.3334 |
|  |  | T19C4.1 | 2.3332 |
|  |  | R11G1.2 | 2.3299 |
|  |  | K08E5.3 | 2.3297 |
|  |  | T10H4.10 | 2.324 |
|  |  | ZK637.11 | 2.3158 |
|  |  | C24B9.9.1 | 2.2059 |
|  |  | C14C6.6 | 1.924 |
|  |  | C35D6.5 | 1.8079 |
