# Supplementary material for: ETS-4 Is a Transcriptional Regulator of Life Span in Caenorhabditis elegans
Source: PLoS Genet. 2010 Sep 16;6(9):e1001125. doi: 10.1371/journal.pgen.1001125 (PMC2940738; doi:10.1371/journal.pgen.1001125)
Supplement: Table S4 — Genes with Altered Expression in ets-4(ok165) and ets-4(uz1) Worms Relative to Wild-type Worms. (0.10 MB DOC) [file pgen.1001125.s012.doc]

Table S4. Genes with Altered Expression in *ets-4(ok165)* and *ets-4(uz1)* Worms Relative to Wild-type Worms.

| **Gene** | **Gene Name** | ***ets-4(ok165)* / WT Ratio** | ***daf-16* Genes^a^** | **Aging Genes^b^** |
| --- | --- | --- | --- | --- |
| F21F8.4.1 |  | 0.0545 |  |  |
| F56A4.3 |  | 0.0581 |  |  |
| F15E11.10 | *srbc-15* | 0.0648 |  |  |
| F01G10.3 | *ech-9* | 0.0938 |  | *ech-9* |
| C49D10.4 | *oac-10* | 0.0971 |  |  |
| F22A3.1 | *ets-4* | 0.1217 |  |  |
| Y46C8AL.3 | *clec-70* | 0.1404 |  |  |
| D2063.2 | *oac-12* | 0.2039 |  |  |
| F59D8.1 | *vit-3* | 0.2234 | *vit-3*^1^ |  |
| F59D8.2 | *vit-4* | 0.2374 | *vit-4*^1^ |  |
| C04F6.1 | *vit-5* | 0.2568 | *vit-5*^1^ | *vit-5* |
| C16C4.4 | *math-14* | 0.3012 |  |  |
| F22A3.4 | *ceh-60* | 0.313 |  |  |
| C16C4.15 | *math-10* | 0.3155 |  |  |
| ZK488.4 | *nhr-251* | 0.3241 |  |  |
| R09B5.3 | *cnc-2* | 0.3321 |  | *cnc-2* |
| F21E9.3 | *ttr-37* | 0.3404 |  |  |
| B0218.8 | *clec-52* | 0.3419 | *clec-52*^2^ | *clec-52* |
| ZK816.5 | *dhs-26* | 0.3435 |  |  |
| T09F5.9 | *clec-47* | 0.355 |  |  |
| C30G12.2 |  | 0.3567 |  | C30G12.2 |
| F42G8.7 |  | 0.3627 |  |  |
| C09B8.4 |  | 0.3647 |  |  |
| F28B12.2 | *egl-44* | 0.3665 |  |  |
| F15E6.4 |  | 0.3739 | F15E6.4^2^ | F15E6.4 |
| C42D4.2 |  | 0.3823 |  |  |
| C16C4.5 | *math-15* | 0.3841 |  |  |
| C23H5.3 | *xbx-4* | 0.3918 |  |  |
| C01B4.8 |  | 0.3937 |  |  |
| Y19D10A.4 |  | 0.4002 |  |  |
| T08A9.7 | *spp-3* | 0.4078 |  | *spp-3* |
| F46H5.8 | *lact-1* | 0.4185 |  |  |
| C17C3.12c.1 | *acdh-2* | 0.424 |  |  |
| R13H4.8 |  | 0.426 |  |  |
| Y57G11B.5 |  | 0.432 |  | Y57G11B.5 |
| C42D8.2 | *vit-2* | 0.4379 | *vit-2*^1^ | *vit-2* |
| C48B4.1 |  | 0.4382 |  | C48B4.1 |
| C54C8.2 |  | 0.4513 |  |  |
| F15B9.1 | *far-3* | 0.4541 | *far-3*^2^ | *far-3* |
| C02A12.4 | *lys-7* | 5.522 | *lys-7*^2^ | *lys-7* |
| H16D19.1 | *clec-13* | 5.3963 | *clec-13*^2^ |  |
| D1014.7 |  | 4.8928 |  |  |
| F28D1.4 | *thn-3* | 3.8969 |  |  |
| D1014.6 |  | 3.8787 |  |  |
| T23F1.5 |  | 3.7753 |  |  |
| Y25C1A.11 | *srg-23* | 3.7685 |  |  |
| F17E9.11 | *lys-10* | 3.6771 |  |  |
| C01G10.15 |  | 3.6458 |  |  |
| F55G11.4 |  | 3.6239 |  | F55G11.4 |
| F10G2.3 | *clec-7* | 3.4655 |  |  |
| T22H6.5 | *abf-5* | 3.4371 |  |  |
| C14C6.2 |  | 3.4294 |  |  |
| F28D1.3 | *thn-1* | 3.3188 | *thn-1*^2^ | *thn-1* |
| E03H4.10 | *clec-17* | 3.2626 |  |  |
| F47H4.2 |  | 3.1877 |  |  |
| ZK666.7 | *clec-61* | 3.1757 |  |  |
| F28D1.5 | *thn-2* | 3.1686 | *thn-2*^2^ | *thn-2* |
| Y59E9AR.6 | *thn-7* | 3.1618 |  |  |
| B0365.6 | *clec-41* | 3.0377 | *clec-41*^1^ | *clec-41* |
| Y73F4A.3.1 |  | 2.956 |  |  |
| F21C10.8a | *pqn-31* | 2.8185 |  |  |
| R13H4.3 | *pho-8* | 2.6947 | *pho-8*^1^ |  |
| C01G10.4 |  | 2.6855 |  | C01G10.4 |
| R11G11.7 | *pqn-60* | 2.6542 |  |  |
| T15D6.11 |  | 2.6509 |  |  |
| C32H11.13 | *dct-19* | 2.598 | *dct-19*^1^ |  |
| F14F8.8 |  | 2.585 |  |  |
| E03H4.4 |  | 2.5536 |  |  |
| T15D6.8 |  | 2.5224 |  |  |
| ZK218.5 |  | 2.3983 |  |  |
| T23F4.3 |  | 2.3532 |  |  |

^a^Murphy et al., 2003.

^b^Budovskaya et al., 2008.

^1^Genes down-regulated in *daf-2* pathway mutant animals but up-regulated in *daf-16(RNAi)*; *daf-2(RNAi)* animals (Murphy et al., 2003).

^2^Genes up-regulated in *daf-2* pathway mutant animals but down-regulated in *daf-16(RNAi)*; *daf-2(RNAi)* animals (Murphy et al., 2003).
