# Supplementary material for: ETS-4 Is a Transcriptional Regulator of Life Span in Caenorhabditis elegans
Source: PLoS Genet. 2010 Sep 16;6(9):e1001125. doi: 10.1371/journal.pgen.1001125 (PMC2940738; doi:10.1371/journal.pgen.1001125)
Supplement: Table S5 — Position of Conserved ETS Binding Motifs in the Transcriptional Control Regions of ETS-4-Regulated Genes. (0.17 MB DOC) [file pgen.1001125.s013.doc]

Table S5. Position of Conserved ETS Binding Motifs^a,b^ in the Transcriptional Control Regions of ETS-4-Regulated Genes^c^.

| **Gene** | **LG**^d^ | **Strand**^e^ | ***ets-4(ok165)* /WT Ratio** | **CDS**  **Start**^f^ | **ETS**  **Motifs**^g^ | **Position of ETS Motifs**^h^ |
| --- | --- | --- | --- | --- | --- | --- |
| F21F8.4 | V | + | 0.05 | 8270623 | 7 | -1427, -1345, -1322, -506, +294, +299, +452, |
| F56A4.3 | V | + | 0.06 | 2453426 | 2 | -535, +99 |
| F15E11.10 | V | + | 0.06 | 2345675 | 0 |  |
| F01G10.3 | IV | - | 0.09 | 10235281 | 3 | -231, +15, +43 |
| C49D10.4 | II | + | 0.10 | 3869893 | 3 | +86, +159, +166 |
| Y46C8AL.3 | IV | + | 0.14 | 3932726 | 0 |  |
| D2063.2 | V | - | 0.20 | 4332536 | 1 | +34 |
| F59D8.1 | X | - | 0.22 | 3572528 | 1 | +330 |
| F59D8.2 | X | - | 0.24 | 3563953 | 1 | +330 |
| C04F6.1 | X | + | 0.26 | 3403468 | 2 | -938, +324 |
| C16C4.4 | II | + | 0.30 | 1869588 | 0 |  |
| F22A3.4 | X | - | 0.31 | 6530105^*^ | 1 | -547^*^ |
| C16C4.15 | II | + | 0.32 | 1871549 | 0 |  |
| ZK488.4 | V | + | 0.32 | 600595 | 1 | +387 |
| R09B5.3 | V | - | 0.33 | 1450175 | 2 | +273, +303 |
| F21E9.3 | X | - | 0.34 | 1336901 | 0 |  |
| B0218.8 | IV | - | 0.34 | 8168734 | 3 | -1069, -182, -115 |
| ZK816.5 | X | - | 0.34 | 3362334 | 1 | +134 |
| T09F5.9 | V | + | 0.36 | 15175737 | 1 | -1192 |
| C30G12.2 | II | + | 0.36 | 7290398 | 4 | -1498, -1249, -877, +15 |
| F42G8.7 | IV | + | 0.36 | 8125634 | 2 | +75, +392 |
| C09B8.4 | X | - | 0.37 | 6019893 | 3 | -1043, +66, +105 |
| F28B12.2a | II | + | 0.37 | 5933501 | 2 | -1494, -1323 |
| F15E6.4 | IV | + | 0.37 | 4291481 | 3 | -1132, -1114, +213 |
| C42D4.2 | IV | + | 0.38 | 7180828 | 8 | -1682, -1580, -1398, -1365, -1085, -796, -791, -13 |
| C16C4.5 | II | + | 0.38 | 1866393 | 0 |  |
| C23H5.3 | IV | + | 0.39 | 2114671 | 0 |  |
| C01B4.8 | V | + | 0.39 | 2490511 | 1 | +63 |
| Y19D10A.4 | V | + | 0.40 | 2385955 | 3 | -266, +244, +349 |
| T08A9.7 | X | - | 0.41 | 7315297 | 7 | -1272, -1258, -1153, -779, -211, +17, +305, |
| F46H5.8 | X | - | 0.42 | 7260326 | 3 | -776, +420, +443 |
| C17C3.12a | II | - | 0.42 | 5547462 | 1 | +389 |
| R13H4.8 | V | + | 0.43 | 11839518 | 13 | -1429, -1444, -1240, -1167, -1125, -883, -268, -79, -72, +66, +125, +200, +426 |
| Y57G11B.5 | IV | + | 0.43 | 14574789 | 2 | -1050, -849 |
| C42D8.2a.1 | X | + | 0.44 | 5101011 | 6 | -1187, -1104, -1011, -124, +110, +379 |
| C48B4.1 | III | - | 0.44 | 9592084 | 1 | -1140 |
| C54C8.2 | I | + | 0.45 | 12444765 | 2 | -1381, +32, |
| F15B9.1 | V | - | 0.45 | 12997492 | 5 | -1150, -210, -162, -154, +184 |
| C02A12.4 | V | - | 5.52 | 3482597 | 5 | -648, +411, +433, +439, +441 |
| H16D19.1 | I | + | 5.40 | 12635160 | 0 |  |
| D1014.7 | V | + | 4.89 | 8118255 | 4 | -1448, -1223, -949, -460 |
| F28D1.4 | IV | + | 3.90 | 12381559 | 19 | -1372, -1294, -1273, -1264, -1071, -1061, -1029, -1000, -927, -879, -832, +116, +194, +215, +245, +411, +447, +453, +482 |
| D1014.6 | V | + | 3.88 | 8121220 | 3 | -1273, -177, -172 |
| T23F1.5 | V | - | 3.78 | 15464395 | 3 | -253, -112, +78 |
| Y25C1A.11 | II | - | 3.77 | 3094798 | 1 | -1195 |
| F17E9.11 | IV | + | 3.68 | 8332807 | 5 | -1155, -1047, -62, +269, +279 |
| C01G10.15 | V | - | 3.65 | 15087272 | 10 | -931, -886, -856, -847, -838, -766, -730, +159, +240, +312 |
| F55G11.4 | IV | + | 3.62 | 12973627 | 2 | +53, +322 |
| F10G2.3 | V | + | 3.47 | 7328396 | 2 | -860, -855 |
| T22H6.5 | X | + | 3.44 | 12792784 | 4 | -990, -986, -382, +290 |
| C14C6.2 | V | + | 3.43 | 563200 | 0 |  |
| F28D1.3 | IV | + | 3.32 | 12380071 | 8 | +116,+194, +215, +224, +417, +427, +459, +488 |
| E03H4.10 | I | - | 3.26 | 12431441 | 0 |  |
| F47H4.2 | V | + | 3.19 | 17353340 | 0 |  |
| ZK666.7 | II | - | 3.18 | 10490412 | 6 | -1514, -1356, -1296, -93, +139, +186 |
| F28D1.5 | IV | + | 3.17 | 12383139 | 19 | -1464, -1386, -1365, -1335, -1169, -1133, -1127, -1098, -1081, -1049, -362, -358, +116, +194, +215, +224, +412, +454, +483 |
| Y59E9AR.6 | IV | - | 3.16 | 5239499 | 0 |  |
| B0365.6 | V | - | 3.04 | 13138039 | 3 | -1316, -1302, +63 |
| Y73F4A.3 | IV | - | 2.96 | 9040440 | 0 |  |
| F21C10.8a | V | + | 2.82 | 9109596 | 5 | -1262, -1133, -699, +421, +463 |
| R13H4.3 | V | + | 2.70 | 11833624 | 4 | -1092, -992, -299, -136 |
| C01G10.4 | V | + | 2.69 | 15089730 | 3 | -604, -544, +230 |
| R11G11.7 | V | + | 2.65 | 504039 | 3 | -1425, -856, +251 |
| T15D6.11 | I | - | 2.65 | 12398281 | 1 | +146 |
| C32H11.13 | IV | + | 2.60 | 12942687 | 0 |  |
| F14F8.8 | V | - | 2.59 | 16672134 | 0 |  |
| E03H4.4 | I | + | 2.55 | 12407552 | 3 | -1398, -954, +176 |
| T15D6.8 | I | + | 2.52 | 12389748 | 2 | -1005, +137 |
| ZK218.5 | V | + | 2.40 | 17101100 | 0 |  |
| T23F4.3 | II | - | 2.35 | 1172090 | 7 | -1056, -908, -897, +138, +192, +238, +253 |

^a^Position specific weight matrix (PWM) used to search for ETS binding motifs


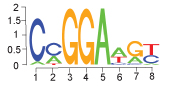


^b^Conservation in *C. elegans*, *C. briggsae*, *C. remanei*, *C. brenneri*, *C. japonica* and *P. pacificus*.

^c^70 genes with altered expression in *ets-4(ok165)* and *ets-4(uz1)* worms relative to wild-type worms.

^d^Linkage group; ^e^Gene transcript orientation; ^f^Coding sequence start

^g^Number of conserved ETS binding motifs

^h^Position of conserved ETS binding motif relative to the CDS start +1 except for F22A3.4 which is relative to the transcription start (denoted by ^*^).
