# Supplementary material for: ETS-4 Is a Transcriptional Regulator of Life Span in Caenorhabditis elegans
Source: PLoS Genet. 2010 Sep 16;6(9):e1001125. doi: 10.1371/journal.pgen.1001125 (PMC2940738; doi:10.1371/journal.pgen.1001125)
Supplement: Table S6 — Oligonucleotide primers used in RT-PCR analyses. (0.04 MB DOC) [file pgen.1001125.s014.doc]

Table S6. Oligonucleotide primers used in RT-PCR analyses.

| Gene | RT-Primer | Forward-Primer | Reverse-Primer |
| --- | --- | --- | --- |
| *ceh-60* | CGTCGATCGGTCTAAAATCGCGA | TCCGTTGGAAGAGGTCAACACTG | CGCTATTAGTCAGGGCTTTTTCC |
| *ech-9* | GGCATTTTGAGAAACTCGACG | ATGACTGTTGCCGATATGAATGG | CAGTGTCATTTTCTTTCCGTTGGG |
| *fat-7* | GTAGTCTTGTGGGAATGTGTGGT | CTGGAAGGAGACAGCATTCATTG | CTGTGGTAAAGACGTTCTCAACG |
| *vit-3* | GTTGACCTCAGCCTGGTCTCCTT | TCTGAGACTCGCTCTAAGGTCAC | GAGTAGACGATCTCCTCCTTTGG |
| *vit-4* | GCTCGTGCTCTTGAATTTGACGG | AGAGGTTGTCAAGTCTGAGACTC | CGATAGCCTTGAATGGGTTGAC |
| *vit-5* | CTCAACCTTCTGCTCCATCTCGATAG | GCAAGAATCTGAGGTTCATTCCG | CTCAACCTTCTGCTCCATCTCGA |
| *vit-2* | GAGTCTCGTGGATCTCCTTAAG | actgtcaacgtcaacggacaag | CTGGAAGATTGTATCCGTCGAC |
| *thn-1* | gtactctgccattcggcatcag | gaatagtgacgcaagagctggtg | cagtgaagtcattgcggagc |
| *lys-7* | gtactctgccattcggcatcag | gaatagtgacgcaagagctggtg | attcagtgaagtcattgcggagc |
| *cdc-42** |  | CTGCTGGACAGGAAGATTACG | CTCGGACATTCTCGAATGAAG |
| *pmp-3** |  | GTTCCCGTGTTCATCACTCAT | ACACCGTCGAGAAGCTGTAGA |

* Reverse-Primers were used as RT-Primer for these genes (Hoogewijs et al., 2008).
