# Supplementary material for: ETS-4 Is a Transcriptional Regulator of Life Span in Caenorhabditis elegans
Source: PLoS Genet. 2010 Sep 16;6(9):e1001125. doi: 10.1371/journal.pgen.1001125 (PMC2940738; doi:10.1371/journal.pgen.1001125)
Supplement: Text S1 — Supplemental Materials and Methods. (0.07 MB DOC) [file pgen.1001125.s015.doc]

**Text S1**

**ETS-4 Is a Transcriptional Regulator of Life Span in *Caenorhabditis elegans***

Bargavi Thyagarajan, Adam G. Blaszczak, Katherine J. Chandler, Jennifer L. Watts, W. Evan Johnson and Barbara J. Graves

**Supplemental Materials and Methods**

**Expression and Purification of ETS-4**

Gateway-adapted expression vector pEXP1-DEST^TM^ (Invitrogen) containing the predicted *ets-4* open reading frame with N-terminal 6XHis and FLAG tag sequences was transformed into *E. coli* strain BL21λ (DE3);pLysS. Expression of ETS-4 protein in cultured cells was induced at midlog phase with 0.5 mM IPTG for 3 hr at room temperature. Bacterial pellets from 400 ml cultures were resuspended in 8 ml of lysis buffer [50 mM sodium phosphate buffer, pH 8.0, 500 mM NaCl, 5 mM imidazole, 10% glycerol] with 1 mM PMSF and lysed by sonication (Heat Systems, Inc). The insoluble fraction was collected by centrifugation at 27,000g for 15 min. To wash the pellet, sonication and centrifugation steps were repeated. The insoluble pellet was solubilized by sonication in 10 ml of urea lysis buffer [50 mM sodium phosphate buffer, pH 8.0, 6 M urea, 500 mM NaCl, 5 mM imidazole, 10% glycerol] with 1 mM PMSF, and then centrifuged at 27,000g for 15 min. The 6XHis tagged ETS-4 was purified from the supernatant at 4°C on a 1 mL HiTrap chelating column (Amersham-Pharmacia). The eluate fractions containing ETS-4 were subjected to dialysis at 4°C, two times for 2 hr each against 10 volumes of TGEK_500_ [20 mM Tris-Cl at pH 7.9, 10% glycerol, 0.1 mM EDTA, 500 mM KCl] with 1 mM DTT and 0.2 mM PMSF. Concentration of the purified protein was estimated on a coomassie stained gel in comparison to known concentrations of bovine serum albumin.

**DNA-binding Assays**

Complementary strands of oligonucleotides for the DNA duplexes used in this assay were 5’ end-labeled with T4 polynuclotide kinase and [γ-^32^ P]ATP (4500 Ci/mol) and annealed as described previously [1].

The high-affinity ETS binding site duplex ETS Site-WT or the mutant ETS binding site duplex, termed ETS Site-MT, were used as probes in the assays. The sequences for the probes were: 5'- TCGACGGCCAAGCCGGAAGTGAGTGCC- 3’ or

5'- TCGACGGCCAAGCCGGATGTGAGTGCC- 3’ (ETS Site-WT) and

5'-TCGACGGCCAAGCCTTAAGTGAGTGCC-3' or

5'- TCGACGGCCAAGCCTTATGTGAGTGCC- 3’ (ETS Site-MT). DNA-binding was assessed by EMSA, performed as described previously [1]. In brief, binding reactions in a 20 μl volume with 25 mM Tris-Cl (pH 7.9), 10% glycerol, 0.1 mM EDTA, 60 mM KCl, 6 mM MgCl_2_, 0.2 mg/ml BSA, 10 mM DTT, and 50 ng of poly[d(I-C)] were incubated for 1 hr at 4°C. EMSA was performed at 4°C on 6% native polyacrylamide gels.

**GFP Reporter Construction and Generation of Transgenic Lines**

5.3 kb of the *ets-4* gene promoter alone or with genomic DNA encompassing the coding sequence for the ETS-4 protein was amplified by PCR and cloned into *PstI/XmaI* sites of pPD95.67 vector (kind gift of Andrew Fire, Stanford University). *Pets-4::gfp* lines were generated by injecting *pha-1(e2123ts);him-8(e1489)* strain [2] with 30 ng/µl of *pha-1(+)*, 10 ng/µl of *ets-4p::GFP* plasmid linearized by *PstI* digestion and 60 ng/µl of carrier DNA. The transgenic strains were selected at 25°C. The *ets-4::gfp* lines were generated by injecting N2 worms with 1 ng/µl or less of *ets-4::gfp* construct linearized by *PstI* digestion, 30 ng/µl of *rol-6(su1006)* plasmid (pRF4) and 70 ng/µl of carrier DNA. Stable *ets-4::gfp* lines could not be maintained for more than a few generations.

Rescue constructs were made by cloning *yfp*::*ets-4*(cDNA) fusion product (created by two-step PCR) into pPD49.26 vector (kind gift of Andrew Fire, Stanford University) using unique *KpnI/EcoRV* sites. Tissue-specific expression was achieved by cloning 4.4 kb of *rab-3* promoter (pan-neuronal expression) and 1.9 kb of *gly-19* promoter (intestinal expression) upstream of *yfp::ets-4*(cDNA) sequence using *HindIII/BamH1* sites. Transgenic lines were generated by injecting *ets-4(ok165);lin-15(n765ts)* strain with 50 ng/µl of *lin-15(+)*, 5 ng/µl of linearized *Pgly-19::yfp::ets-4* or *Prab-3::yfp::ets-4* plasmid and 50 ng/µl of carrier DNA. Transgenic lines were maintained by picking non-Muv hermaphrodites. *ets-4(ok165)* Control refers to injection control strains and were created by injecting *ets-4(ok165);lin-15(n765ts)* strain with 50 ng/µl of *lin-15(+)* and 50 ng/µl of carrier DNA and subsequently maintaining transgenic lines by picking non-Muv hermaphrodites.

**Fatty Acid and Lipid Analysis**

Fatty acid and lipid analysis was performed as described previously [3]. Briefly, synchronized populations of young adult nematodes grown on plates with OP50 bacteria were used for the analysis. Chloroform-methanol extraction was performed to extract total lipids later subjected to gas chromatography. A measure of total lipids was obtained and normalized to total protein levels determined by Bradford assay. Next, the lipid extracts were subjected to thin-layer chromatography to separate phospholipids (PL) from the triacyglycerides (TAG). Fatty acid extracts were subjected to gas chromatography to determine percentage of individual fatty acids in the TAG and PL fractions.

**Feeding Behavior Analysis**

Animals were grown at 20°C and scored at young adult stage. Defecation rates were determined as described previously [4]. A cycle period was defined as the interval between two successive posterior body-wall muscle contractions (pBoc). Pharyngeal pumping rate was assessed as described previously [5]. Briefly, pumping rates were determined using a dissecting microscope by counting pumps of the terminal pharyngeal bulb for one-minute intervals.

**Stress Assays**

Heat stress assays were performed as described before [6]. Gravid worms were allowed to lay eggs for 3-4 hr to obtain synchronous progeny. The progeny were allowed to develop at 15°C and shifted to 25°C after L3 larval stage to avoid the constitutive dauer arrest phenotype associated with *daf-2(e1370)* mutant animals. Synchronous day 2 adult hermaphrodites were shifted to 35°C and survival was monitored. Triplicate plates for each strain were scored for each time point. Oxidative stress assays were performed as described previously [7]. Synchronous day 2 adult hermaphrodites were transferred to Paraquat (methylviologene, Sigma-Aldrich) containing plates (90 µl of 150 µM Paraquat added on top of NGM plates already seeded with OP50 bacteria). Survival was monitored every day. At least three independent repeats were conducted.

**GFP Quantification**

Quantification of GFP expression was done as described previously [8]. Briefly, GFP fluorescence of at least 20 age-matched (day 1 of adulthood) worms per strain was measured. Images were acquired using identical exposure times and settings. Quantification of mean fluorescence was carried out using the ImageJ (version 1.36b), a public domain image processing program.

**Searching for Conserved Binding Motifs**

The DNA sequence of the region from 1500 base pairs (bp) upstream of to 500 bp downstream from the transcription start site of ETS-4-regulated genes were obtained from the UCSC genome browser (http://genome.ucsc.edu/). Conservation scores were downloaded from the UCSC genome browser, originating from the multiple alignment between six nematode species, *C. elegans, C. remanei, C. briggsae, C. brenneri, C. japonica, P. pacificus*, using the MULTIZ alignment algorithm [9] and then scoring the alignment using the phastCons algorithm [10] to obtain conservation values between 0 and 1. Position specific weight matrix (PWM) to represent and search for ETS binding motifs were obtained from previous studies [11]. Searching with motif derived for SPDEF (the mammalian orthology of ETS-4) yielded similar results [12].Motifs were scored using the following approach. Let **y** represent a candidate sequence for the motif and let *y_i_* be the *i*th nucleotide in the sequence **y**. Denote the *ij*th element of the L by 4, PWM for the motif be denoted by *p_i_*_j_, and let *c_i_* be the genome conservation of *y_i_* across the 6 nematode genomes. The motif score, *f*(**y**), is calculated by

,

where *I*(*y_i_=j*)=1 if *y_i_* is equal to nucleotide *j*, for *j*∈[*A,C,G,T*], and *I*(*y_i_=j*)=0 otherwise. Therefore, the motif score *f*(**y**) represents a combination of the DNA conservation across nematode species and as well as degree to which the sequence matches the motif of interest. Motifs were selected using a cutoff that simultaneously requires the genome conservation to be reasonably high (>0.75) and that sequence is much more likely to be a motif than a random background (>10 times).

**References**

1. Jonsen MD, Petersen JM, Xu Q, Graves BJ (1996) Characterization of the cooperative function of inhibitory sequences of Ets-1. Mol Cell Biol 16: 2065-2073.

2. Hodgkin J, Horvitz HR, Brenner S (1979) Nondisjunction Mutants of the Nematode CAENORHABDITIS ELEGANS. Genetics 91: 67-94.

3. Brock TJ, Browse J, Watts JL (2006) Genetic regulation of unsaturated fatty acid composition in C. elegans. PLoS Genet 2: e108.

4. Iwasaki K, Liu DW, Thomas JH (1995) Genes that control a temperature-compensated ultradian clock in Caenorhabditis elegans. Proc Natl Acad Sci U S A 92: 10317-10321.

5. Panowski SH, Wolff S, Aguilaniu H, Durieux J, Dillin A (2007) PHA-4/Foxa mediates diet-restriction-induced longevity of C. elegans. Nature 447: 550-555.

6. Lithgow GJ, White TM, Melov S, Johnson TE (1995) Thermotolerance and extended life-span conferred by single-gene mutations and induced by thermal stress. Proc Natl Acad Sci U S A 92: 7540-7544.

7. Masse I, Molin L, Mouchiroud L, Vanhems P, Palladino F, et al. (2008) A novel role for the SMG-1 kinase in lifespan and oxidative stress resistance in Caenorhabditis elegans. PLoS One 3: e3354.

8. Budovskaya YV, Wu K, Southworth LK, Jiang M, Tedesco P, et al. (2008) An elt-3/elt-5/elt-6 GATA transcription circuit guides aging in C. elegans. Cell 134: 291-303.

9. Blanchette M, Kent WJ, Riemer C, Elnitski L, Smit AF, et al. (2004) Aligning multiple genomic sequences with the threaded blockset aligner. Genome Res 14: 708-715.

10. Siepel A, Bejerano G, Pedersen JS, Hinrichs AS, Hou M, et al. (2005) Evolutionarily conserved elements in vertebrate, insect, worm, and yeast genomes. Genome Res 15: 1034-1050.

11. Nye JA, Petersen JM, Gunther CV, Jonsen MD, Graves BJ (1992) Interaction of murine Ets-1 with GGA-binding sites establishes the ETS domain as a new DNA-binding motif. Genes Dev 6: 975-990.

12. Wei GH, Badis G, Berger MF, Kivioja T, Palin K, et al. Genome-wide analysis of ETS-family DNA-binding in vitro and in vivo. Embo J.
